# MYCN drives oncogenesis by cooperating with the histone methyltransferase G9a and the WDR5 adaptor to orchestrate global gene transcription

**DOI:** 10.1371/journal.pbio.3002240

**Published:** 2024-03-28

**Authors:** Zhihui Liu, Xiyuan Zhang, Man Xu, Jason J. Hong, Amanda Ciardiello, Haiyan Lei, Jack F. Shern, Carol J. Thiele

**Affiliations:** Pediatric Oncology Branch, National Cancer Institute, Bethesda, Maryland, United States of America; B.C. Cancer Agency, CANADA

## Abstract

MYCN activates canonical MYC targets involved in ribosome biogenesis, protein synthesis, and represses neuronal differentiation genes to drive oncogenesis in neuroblastoma (NB). How MYCN orchestrates global gene expression remains incompletely understood. Our study finds that MYCN binds promoters to up-regulate canonical MYC targets but binds to both enhancers and promoters to repress differentiation genes. MYCN binding also increases H3K4me3 and H3K27ac on canonical MYC target promoters and decreases H3K27ac on neuronal differentiation gene enhancers and promoters. WDR5 facilitates MYCN promoter binding to activate canonical MYC target genes, whereas MYCN recruits G9a to enhancers to repress neuronal differentiation genes. Targeting both MYCN’s active and repressive transcriptional activities using both WDR5 and G9a inhibitors synergistically suppresses NB growth. We demonstrate that MYCN cooperates with WDR5 and G9a to orchestrate global gene transcription. The targeting of both these cofactors is a novel therapeutic strategy to indirectly target the oncogenic activity of *MYCN*.

## Abstract 2

The oncoprotein MYCN drives oncogenesis in neuroblastoma by activating the expression of genes involved in ribosome biogenesis and protein synthesis, while repressing neuronal differentiation genes, but the underlying mechanisms remain unknown. This study shows that MYCN cooperates with the histone methyltransferase G9a and the WDR5 adaptor to orchestrate global gene transcription.

## Introduction

The deregulation of *MYC* family oncogenes including *c-MYC*, *MYCN*, and *MYCL* occurs in most cancers and frequently marks those associated with poor prognosis [1–5]. *MYCN* is implicated in many pediatric embryonal tumors such as neuroblastoma (NB), rhabdomyosarcoma, medulloblastoma, and more recently in therapy-resistant adult cancers including subtypes of breast cancer and prostate cancers [2,5]. *MYCN* encodes a basic helix-loop-helix-leucine zipper transcription factor (TF) named N-Myc or MYCN and exhibits high-structural homology with c-MYC [2]. The c-MYC TF directly regulates gene transcription controlling cell growth, cell cycle progression, ribosome biogenesis, protein synthesis, genomic stability, glucose and nucleotide metabolism, apoptosis, etc. [1,3]. Additionally, c-MYC inhibits differentiation in normal hematopoietic, mesenchymal, adipocytic, neuronal, and muscle cells, as well as in cancer cells such as pheochromocytoma and erythroleukemia [6–8]. Some studies showed that c-MYC represses cell differentiation by blocking or repressing the expression of differentiation-inducing genes [6]. Early studies indicated that *MYCN* overexpression in NB cells leads to a transcriptome enriched in canonical *MYC* target genes including genes involved in ribosome biogenesis and protein synthesis [9,10]. Later, the identification of a functional MYCN signature gene set in one NB cell line indicated that MYCN suppresses genes associated with neuronal differentiation [11]. However, the molecular mechanisms by which MYCN orchestrates these global gene expression changes at a genome-wide level remain unclear.

Most eukaryotic TFs act by recruiting coactivators, or corepressors, which include chromatin remodeling complexes and covalent histone-modifying complexes [12]. Only a handful of coactivators and corepressors of MYCN have been experimentally demonstrated to mediate its transcriptional activity, and in these studies only a few target genes have been assessed [5,13–18]. However, the cooperation between MYCN and its cofactors has not been systematically investigated on a genome-wide level. *MYCN* is a bona fide oncogenic driver in NB [5,19–21] and it is known that the silencing of *MYCN* results in a decrease in cell proliferation and induction of cell differentiation in NB cells [22,23]. The therapeutic aim of directly targeting MYCN remains challenging due to its structural flexibility. However, one can target MYCN indirectly by identifying the enzymatically active cofactors that mediate MYCN regulated oncogenic transcriptional programs. This requires a genome-wide understanding of the critical cofactors by which MYCN regulates global gene expression.

To investigate how MYCN globally regulates gene transcription, we combined a protein interactome assay and genome-wide approaches including RNA sequencing (RNA-seq) and chromatin immunoprecipitation followed by DNA sequencing (ChIP-seq). Our study demonstrates that WDR5 assists MYCN to bind promoters to activate canonical MYC targets, whereas MYCN recruits G9a to enhancers to repress neuronal differentiation genes in NB. The simultaneous targeting of both WDR5 and G9a-regulated transcriptional activities is a more effective approach to indirectly target MYCN and its oncogenic program.

## Results

### MYCN governs a malignant NB cell identity by activating canonical MYC target genes and suppressing neuronal differentiation genes

**Fig 1:**
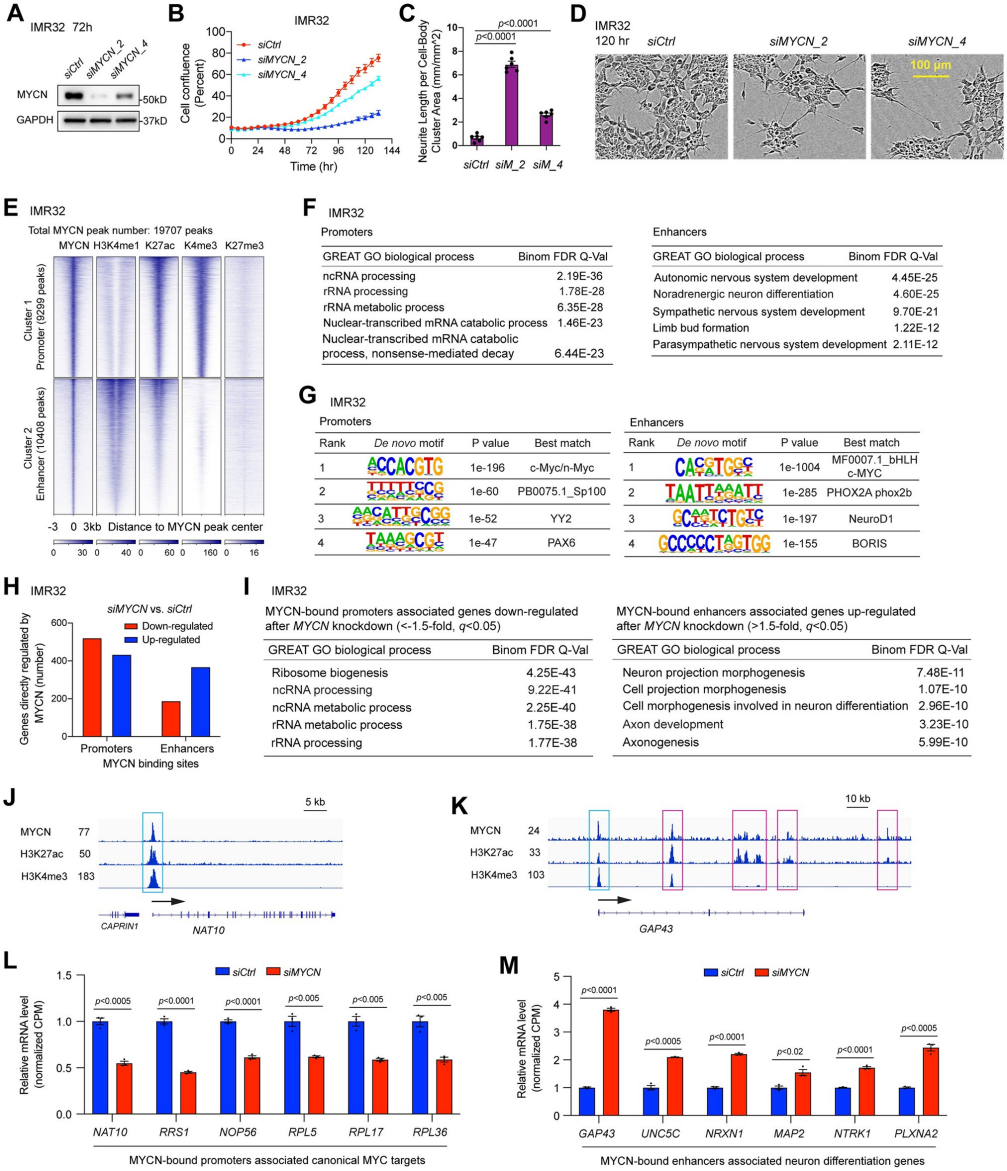
MYCN governs a malignant NB cell identity by directly activating canonical MYC target genes and suppressing neuronal differentiation genes. (**A**) *MYCN* knockdown using 2 different siRNAs in IMR32 cells for 72 h decreases MYCN protein levels as detected by western blot assay. (**B**) *MYCN* knockdown using 2 different siRNAs in IMR32 cells decreases cell number as detected by IncuCyte cell confluence assay. (**C**) and (**D**) *MYCN* knockdown in IMR32 cells increases neurite length as assessed by the IncuCyte neurite analysis assays and phase-contrast images. (**E**) *k*-Means clustering of MYCN and histone marks ChIP-seq around MYCN binding sites of NB cell line IMR32 (±3 kb) shows that MYCN binds to proximal regulatory elements containing active promoters marked by H3K27ac and H3K4me3 signals, and binds distal regulatory elements containing enhancers marked by H3K4me1 and H3K27ac signals. (**F**) GREAT GO analysis indicates that MYCN-bound promoter-associated genes are enriched in RNA processing and MYCN-bound enhancer-associated genes are enriched in nervous system development. (**G**) HOMER motif analysis shows the enrichment of canonical E-box in the promoters and the enrichment of non-canonical E-box in the enhancers. (H) The combination of RNA-seq and ChIP-seq data analysis in IMR32 cells after the silencing of *MYCN* shows that more MYCN-bound promoter-associated genes are down-regulated (521 vs. 434) while more MYCN-bound enhancer-associated genes are up-regulated (368 vs. 189) after the silencing of *MYCN*. (I) GREAT GO analysis indicates that MYCN-bound promoter-associated genes down-regulated after *MYCN* knockdown in IMR32 cells are enriched in ribosome biogenesis and RNA processing (left panel), and MYCN-bound enhancer-associated genes up-regulated after *MYCN* knockdown are enriched in neuronal differentiation (right panel). (J) Signal tracks show the MYCN, H3K27ac, and H3K4me3 ChIP-seq signals at the promoter of *NAT10* gene (cyan box). (K) Signal tracks show the MYCN, H3K27ac, and H3K4me3 ChIP-seq signals at the promoter (cyan box) and enhancers (pink box) of *GAP43* gene. (L) The silencing of *MYCN* in IMR32 cells results in a significant down-regulation of genes involved in ribosome formation based on the RNA-seq results, with MYCN binding to the promoters of these genes. The *p*-value is calculated using one-way ANOVA. (I) The silencing of *MYCN* in IMR32 cells results in a significant up-regulation of genes involved in neuron projection morphogenesis based on the RNA-seq results, with MYCN binding to the enhancers of these genes. The indicated *p*-value is calculated using a one-way ANOVA. The data underlying the graphs in the figure are shown in S1 Data. CPM, counts per million; GO, Gene Ontology; GREAT, genomic regions enrichment of annotation tool; NB, neuroblastoma.

We systematically investigated *MYCN* biological functions and transcriptional activity in several NB cell lines through both loss and gain of function studies. As previously reported [22,23], the knockdown of *MYCN* in IMR32 cell line using 2 different siRNAs (*siMYCN_2* and *siMYCN_4*) reproducibly resulted in a decrease in cell proliferation and an increase in neurite extension (Figs 1A–1D and S1A). These consistent outcomes establish that the observed effects of *MYCN* loss of function are not due to off-target effects of the siRNAs. Thus, in subsequent studies, we utilized one of these 2 siRNAs (*siMYCN_2*) in KCNR, LAN5, and BE(2)C cell lines to show that the silencing of *MYCN* resulted in a decrease in cell proliferation and an increase in neurite extension (S1B–S1J Fig). For gain of *MYCN* function studies, the non-tumorigenic SHEP NB cell line that does not express *MYCN* (*MYCN* non-amplified) was used. Overexpression of *MYCN* in SHEP for 2 days resulted in a cell morphology change with a flatter, more round cell bodies compared to control cells (S1K and S1L Fig). Consistent with a previous report [24], an anchorage-independent cell proliferation assay showed that overexpression of *MYCN* in SHEP cells increased soft agar colony formation (S1M and S1N Fig).

To identify MYCN-regulated genes, we conducted RNA-seq experiments after silencing *MYCN* expression. Additionally, we explored published RNA-seq datasets following *MYCN* silencing (GSE183641) [25] (S1 Table). Gene set enrichment analysis (GSEA) [26,27] showed that the silencing of *MYCN* using 2 different siRNAs (*siMYCN_2* or *siMYCN_4*) in IMR32 resulted in a similar negative enrichment of MYC targets and ribosome biogenesis genes, whereas neuron markers and genes that positively regulate synaptic transmission were positively enriched (S1O and S1P Fig). Similar results were observed when *MYCN* was knocked down in other NB cell lines by using *siMYCN_2* (S1Q and S1R Fig), or when MYCN was overexpressed in SHEP cells (S1S Fig). These loss and gain of *MYCN* function studies in multiple NB cell lines indicate that MYCN activates canonical *MYC* target genes and represses neuronal differentiation genes to govern a malignant NB cell identity.

### Genome-wide mapping of MYCN binding

To identify genes directly regulated by MYCN and the chromatin status associated with these genes, we performed ChIP-seq experiment using an MYCN antibody and antibodies that recognize different histone marks in IMR32 cells. High-confidence ChIP-seq peaks were called by MACS2 and peaks were normalized to reads per kilobase per million reads normalized read numbers (RPKM, see Materials and methods for details). Peaks from ChIP-seq of MYCN and histone marks were selected based on *p*-value (all *p* < 10^−7^). The MYCN ChIP-seq heatmap represented genome-wide stringent sets of MYCN peaks (MYCN binding sites, total 19,707 peaks) within the whole genome (Fig 1E). These MYCN peaks were segmented based on their colocalizations with the indicated histone marks (Fig 1E) through *k*-means clustering, revealing a total of 2 distinct clusters of MYCN peaks that colocalize with differing histone marks. In general, H3K4me1 marks both active and poised enhancers, H3K27ac marks both active enhancers and promoters, H3K4me3 marks active promoters, while H3K27me3 marks repressed chromatin. Based on the peak distribution and the signal intensity of the histone marks, cluster 1 of the heatmap represented active promoters or proximal regulatory regions, cluster 2 represented both active enhancers and weak or poised enhancers based on H3K4me1 and H3K27ac signal intensities (Fig 1E). The heatmap showed that MYCN overlapped with active histone marks but not the repressive histone mark H3K27me3 (Fig 1E). Among all the 19,707 MYCN peaks, 47.2% (9,299 peaks) are within promoters and 52.8% (10,408 peaks) are within enhancers. Genomic regions enrichment of annotation tool (GREAT) [28] was used to analyze the peak distribution of each of these clusters. Consistent with being located in promoter and enhancer regions, the results showed that the majority of peaks for cluster 1 were within 5 kb of the transcription start sites (TSSs), whereas the majority of peaks for cluster 2 were over 5 kb from the TSS (S1T Fig). GREAT Gene Ontology (GO) analysis of MYCN binding sites associated genes showed that MYCN-bound promoters (cluster 1) associated genes are enriched in RNA processing, ribosome assembly, metabolic process, protein synthesis, and other processes (Fig 1F, left panel, S2 Table), while MYCN-bound enhancers (cluster 2) associated genes are enriched in development such as top-ranked nervous system development (Fig 1F, right panel). Additionally, GREAT analysis of MYCN-bound active enhancers in IMR32, marked by H3K27ac peaks that do not overlap with H3K4me3 peaks, indicates that these enhancers’ associated genes are involved in regulating nervous system development (top-ranked). Notably, genes related to other developmental processes, such as limb bud formation and arterial endothelial cell differentiation, as well as pathways that are unrelated to differentiation, are also enriched (S2 Table). Furthermore, GREAT analysis indicates that genes associated with all active enhancers, or genes associated with active enhancers with or without MYCN binding are enriched in similar pathways (S2 Table). HOMER motif analysis showed that in IMR32 cells, E-boxes were enriched in MYCN-bound promoters and enhancers (Fig 1G).

We further dissected MYCN binding sites and their associated genes using a publicly available MYCN ChIP-seq dataset generated in another *MYCN*-amplified cell line BE(2)C (GSE94822). Here, we simply separated MYCN binding sites into 2 groups, which include promoter regions (−1 kb–+100 bp from TSS) and distal regulatory regions (the regions outside of the promoter) as annotated by the HOMER tool. GREAT GO analysis of MYCN binding sites associated genes showed that MYCN-bound promoter-associated genes were enriched in canonical MYC target genes that regulate RNA processing and ribosome biogenesis, whereas MYCN-bound distal regulatory regions associated genes were enriched in nervous system development (S1U Fig).

Altogether, the ChIP-seq analyses in both IMR32 and BE(2)C cells indicate that MYCN-bound promoters are associated with canonical MYC target genes and MYCN-bound distal regulatory regions are associated with neuronal genes in NB.

### MYCN binds promoters to activate canonical MYC targets but binds to both enhancers and promoters to repress neuronal differentiation genes in NB

To investigate how DNA bound MYCN affected gene transcription, we performed an integrative analysis of the MYCN ChIP-seq and RNA-seq data in IMR32 cells. A total of 1,823 genes, showing an expression change of >1.5-fold or <−1.5-fold with an adjusted *p*-value of <0.05 detected by RNA-seq upon silencing of *MYCN*, are considered MYCN-regulated genes (S1 Table). Genes associated with MYCN-bound promoters or enhancers (Fig 1E) are determined by using the HOMER peak annotation tool. We compared the list of MYCN-regulated genes identified by RNA-seq with the list of genes associated with MYCN-bound promoters or enhancers. This analysis revealed that among the 1,823 MYCN up- and down-regulated genes, 1,512 were bound by MYCN within the promoters, enhancers, or both. This analysis suggests that these genes are direct targets of MYCN. We found that for MYCN-bound promoter-associated genes, 521 genes (54.6%) were down-regulated and 434 genes (45.4%) were up-regulated after the silencing of *MYCN*. However, for MYCN-bound enhancer-associated genes, a greater number of genes were up-regulated (368 genes, 66.1%) compared to down-regulated genes (189 genes, 33.9%) (Fig 1H). MYCN-bound promoter-associated MYCN-activated genes (whose expression decreased after *MYCN* silencing) were significantly enriched in ribosome biogenesis and RNA processing (Fig 1I, left panel). In contrast, MYCN-bound promoter-associated, MYCN-repressed genes (whose expression increased after *MYCN* silencing) were enriched in pons development and response to axon injury (S1V Fig). The MYCN-bound enhancer-associated MYCN-repressed genes were significantly enriched in neuronal differentiation (Fig 1I, right panel), with MYCN-bound enhancer-associated MYCN-activated genes enriched in chordate embryonic development (S1W Fig). RPKM normalized signal tracks showed an MYCN ChIP-seq peak at the promoter of *NAT10* (N-Acetyltransferase 10), a gene required for ribosome biogenesis (Fig 1J). For the neuronal differentiation gene, *GAP43* (growth associated protein 43), a single MYCN ChIP-seq peak was observed at the promoter while multiple peaks were observed at its enhancers (Fig 1K). We found that in addition to binding to the enhancers of neuronal genes in NB cells, MYCN also binds to the promoters of these genes. The expression changes of representative MYCN-bound promoter-associated ribosome biogenesis genes after MYCN depletion were shown in Fig 1L, and those for representative MYCN-bound enhancer-associated neuronal genes were shown in Fig 1M. These results indicate that MYCN activates canonical MYC target genes mainly through binding to promoters in NB, whereas MYCN binds to both enhancers and promoters to suppress neuronal differentiation genes.

Bona fide MYC-target genes encompass positive regulators of cell cycle progression, such as *CDK4* and *cyclin A*, genes involved in ribosome biogenesis and protein synthesis, as well as those involved in metabolism. Therefore, a cell exhibiting high MYC levels would be primed for active proliferation due to increased cycling activity, larger cell mass, and enhanced competence in energy production [6,29]. In addition to genes related to ribosome biogenesis and protein synthesis, we specifically examined canonical MYC-target genes associated with cell proliferation and metabolism. Our findings indicate that MYCN binds to the promoters, but not enhancers, of these genes in most cases. For example, signal tracks revealed that MYCN colocalizes with H3K27ac and H3K4me3 on the promoters, but not enhancers, of cell cycle genes *CDK4* and *CCNA2*, as well as metabolic genes *ODC1*, *LDHA*, *GLS*, *PRIM1*, and *PKM* (S1X Fig). Consistently, RNA-seq data analysis demonstrated that *MYCN* knockdown results in a negative enrichment of genes involved in cell cycling and metabolism (S1Y and S1Z Fig).

### MYCN binds to promoters to activate canonical MYC targets but binds to enhancers to repress skeletal muscle genes in rhabdomyosarcoma

**Fig 2:**
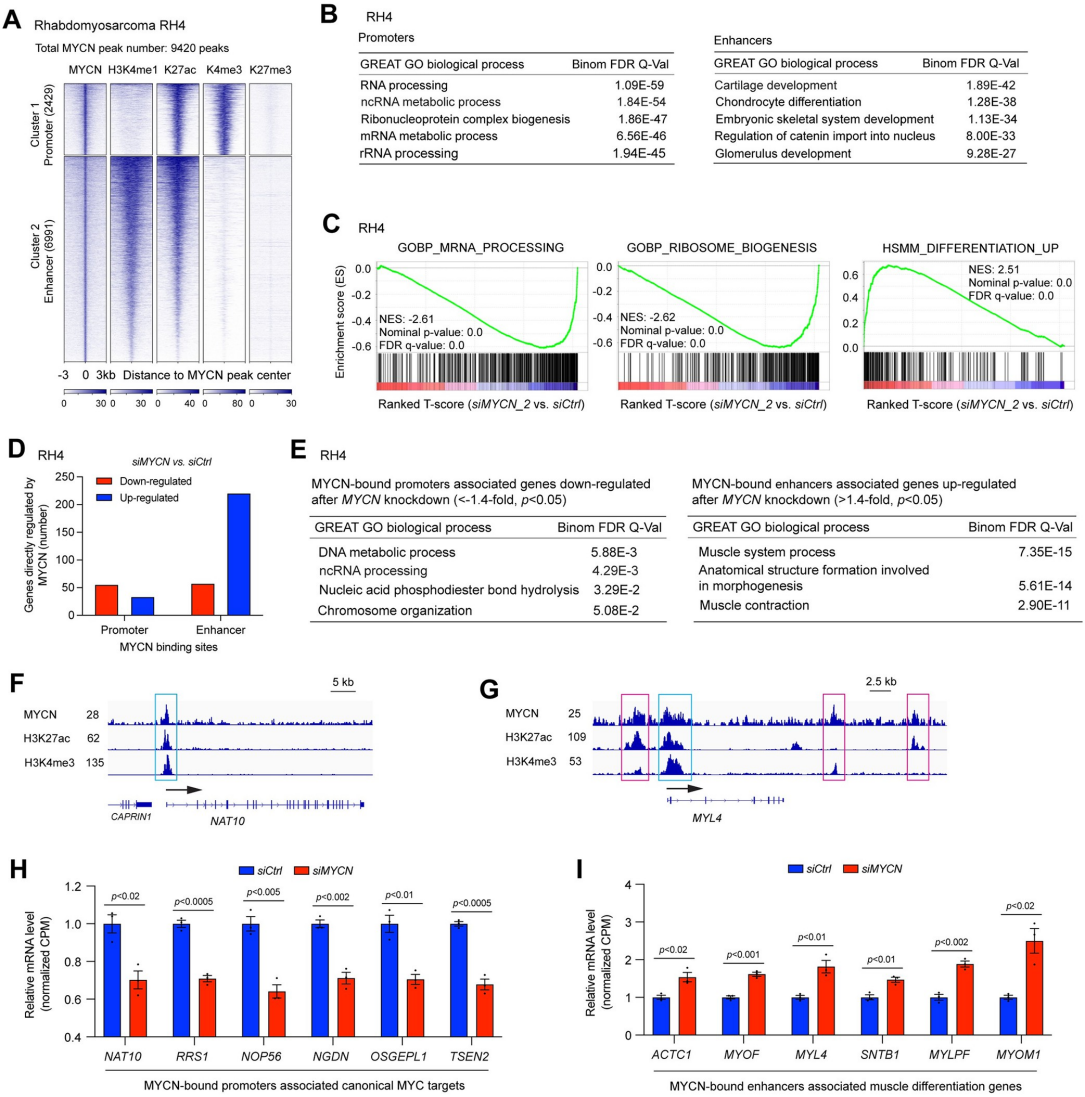
MYCN binds to the promoters to activate canonical MYC targets and binds to the enhancers and promoters to repress muscle differentiation genes in RMS. (**A**) *K*-Means clustering of MYCN and histone marks ChIP-seq around MYCN binding sites of rhabdomyosarcoma cell line RH4 (±3 kb). (**B**) GREAT GO analysis indicates that MYCN-bound promoters associated genes are enriched in RNA processing and MYCN-bound enhancers associated genes are enriched in cartilage system and skeletal system development. (**C**) GSEA of the RNA-seq data shows that *MYCN* knockdown in RH4 cells for 72 h results in a negative enrichment of canonical MYC target genes that are involved in RNA processing and ribosome biogenesis, as well as a positive enrichment of human myogenic differentiation signature genes (HSMM_UP). (**D**) The combination of RNA-seq and ChIP-seq data analysis in RH4 cells shows that more MYCN-bound promoter-associated genes are down-regulated (56 vs. 34) but more MYCN-bound enhancer-associated genes are up-regulated (221 vs. 58). (**E**) GREAT GO analysis indicates that MYCN-bound promoters associated genes down-regulated after *MYCN* knockdown in RH4 cells are enriched in RNA processing (left panel), while MYCN-bound enhancers associated genes up-regulated after the knockdown of *MYCN* are enriched in muscular system process (right panel). (F) Signal tracks show the MYCN, H3K27ac, and H3K4me3 ChIP-seq signals at the promoter of *NAT10* gene (cyan box). (G) Signal tracks show the MYCN, H3K27ac, and H3K4me3 ChIP-seq signals at the promoter (cyan box) and enhancers (pink box) of *MYL4* gene. (H) *MYCN* knockdown in RH4 cells results in a significant down-regulation of genes involved in ribosome formation based on the RNA-seq results, with MYCN binding to the promoters of these genes. The *p*-value indicated is calculated using a one-way ANOVA. (I) *MYCN* knockdown in RH4 cells results in a significant up-regulation of genes involved in muscle system processes based on the RNA-seq results, with MYCN binding to the enhancers of these genes. The *p*-value indicated is calculated using a one-way ANOVA. The data underlying the graphs in the figure are shown in S1 Data. CPM, counts per million; GO, Gene Ontology; GREAT, genomic regions enrichment of annotation tool; GSEA, gene set enrichment analysis; RMS, rhabdomyosarcoma.

To investigate whether the mechanism by which MYCN regulates gene expression in NB is consistent in other embryonal tumors with MYCN overexpression, we interrogated publicly available ChIP-seq data from rhabdomyosarcoma (RMS) cell line RH4 that expresses high levels of *MYCN* (GSE83728) [30]. *K*-means clustering results showed that in RH4 cells, among 9,420 MYCN bound peaks, 2,429 (25%) MYCN peaks were within promoters while 6,991 (75%) of MYCN peaks were within enhancers (Fig 2A). MYCN bound to both active promoters and active enhancers but not to repressed chromatin as indicated by different histone marks (Fig 2A). Interestingly, we found that a higher percentage of MYCN binding sites is within enhancers in RH4 cells compared to IMR32 cells (Figs 1E and 2A). High levels of MYC oncoproteins tend to bind to distal regulatory through enhancer invasion [31–33]. Unexpectedly, western blot results showed that MYCN was more highly expressed in IMR32 than in RH4 cells (S2A Fig). Thus, the higher percentage of MYCN invading enhancers in RH4 cells compared to IMR32 cells might be attributed to the presence of more preestablished open chromatin at the enhancer regions in RH4, facilitated by the existence of other TFs or pioneer factors, allowing MYCN to access them. MYCN-bound promoter-associated genes were enriched in canonical MYC target genes such as genes that regulate RNA processing, ribosome biogenesis, protein synthesis, and metabolic process (Fig 2B, left panel, S3 Table), whereas MYCN-bound enhancer-associated genes were enriched in skeletal system development, cartilage development, as well as pathways that are unrelated to differentiation (Fig 2B, right panel, S3 Table). *MYCN* silencing in RH4 cells inhibited cell proliferation indicated by both the cell confluence assay and cell imaging (S2B–S2D Fig). GSEA of the RNA-seq data (S1 Table) showed that *MYCN* knockdown in RH4 cells resulted in a negative enrichment of canonical MYC target genes that are involved in RNA processing, ribosome biogenesis, cell cycle progression, as well as a positive enrichment of myogenic differentiation signature genes (HSMM_UP) [34] (Figs 2C and S2E). To investigate the association of MYCN binding sites and the expression of their target gene, we performed an integrative analysis of MYCN ChIP-seq and RNA-seq data. We found that more of MYCN-bound enhancer-associated genes were up-regulated (221 versus 58) after *MYCN* silencing in RMS cells (Fig 2D). GO analysis indicated that MYCN-bound promoter-associated genes that were down-regulated after *MYCN* silencing in RH4 cells were enriched in RNA processing (Fig 2E, left panel), whereas MYCN-bound enhancer-associated genes that were up-regulated after *MYCN* silencing were enriched in muscle system processes (Fig 2E, right panel). Neither MYCN-bound promoter-associated MYCN-repressed genes nor MYCN-bound enhancer-associated MYCN-activated genes showed significantly enriched biological processes. Signal tracks showed an MYCN ChIP-seq peak at the promoter of *NAT10* gene that is required for ribosome biogenesis (Fig 2F), while MYCN ChIP-seq peaks were observed both at the promoter and enhancers of a representative muscle gene, *MYL4* (myosin light chain 4) (Fig 2G). Representative MYCN-bound promoter-associated MYCN-activated genes that are involved in ribosome biogenesis were shown in Fig 2H, and representative MYCN-bound enhancer-associated MYCN-repressed genes that are involved in muscle system processes were shown in Fig 2I.

Thus, in 2 distinct pediatric cancers, NB and RMS, our results demonstrate that MYCN directly activates canonical MYC target genes mainly through binding to the promoters of these genes, while repressing tissue-specific differentiation genes mainly through binding to both enhancers and promoters of these genes (Figs 1I–1M and 2E–2I).

### *MYCN* depletion alters histone modifications on its target genes

We next asked whether the activation of canonical MYC target genes and repression of neuronal differentiation genes by MYCN are associated with changes of histone modifications after MYCN depletion in IMR32 cells. To compare the ChIP-seq signal intensity in control and *MYCN* silenced samples, the ChIP-seq peaks were RPKM normalized. While changes in MYCN levels led to decreases in the average MYCN ChIP-seq signals, there were no changes in the average ChIP-seq signals for histone marks globally (S3A Fig). Analysis of MYCN binding focused on whole genome-wide TSS showed similar results (S3B Fig).

**Fig 3:**
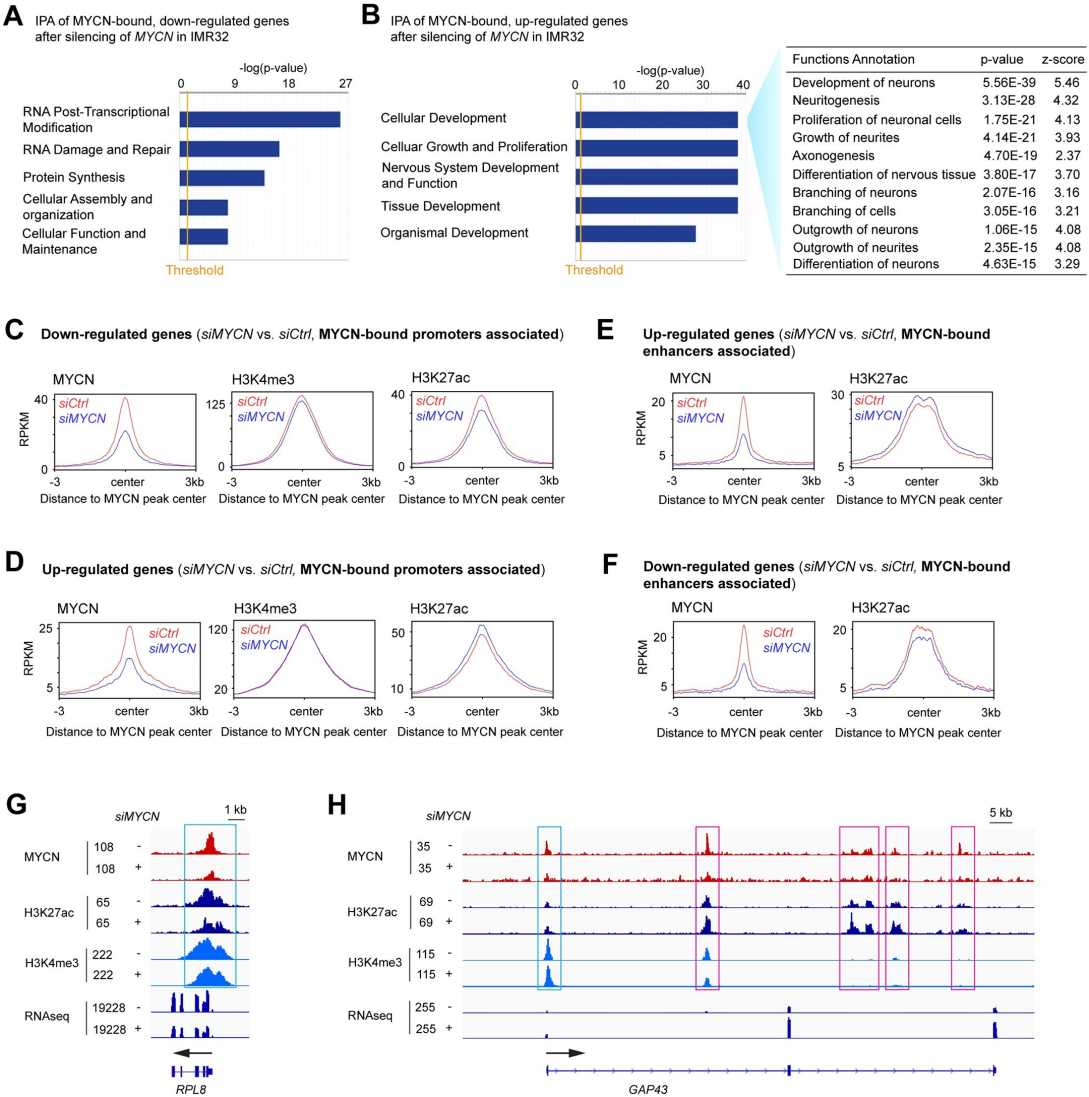
MYCN depletion alters histone modifications on its target genes. (**A**) The integrative analysis of the MYCN ChIP-seq and RNA-seq merged data by IPA shows that the MYCN-bound, down-regulated genes after *MYCN* silencing in IMR32 cells are enriched in RNA posttranscriptional modification and protein synthesis. (**B**) IPA of the RNA-seq data shows that the MYCN-bound up-regulated genes after *MYCN* silencing are positively enriched in neuronal differentiation. (**C**) When focusing on MYCN-bound promoters associated, down-regulated genes, metagene plots show that *MYCN* silencing results in a decrease in the average ChIP-seq signals of H3K4me3 and H3K27ac at the MYCN peak center. (**D**) When focusing on MYCN-bound promoters associated, up-regulated genes, metagene plots show that *MYCN* silencing results in an increase in the average ChIP-seq signal of H3K27ac at the MYCN peak center. (**E**) When focusing on MYCN-bound enhancers associated, up-regulated genes, metagene plots show that *MYCN* silencing results in an increase in the average ChIP-seq signal of H3K27ac at the MYCN peak center. (**F**) When focusing on MYCN-bound enhancers associated, down-regulated genes, metagene plots show that *MYCN* silencing results in a decrease in the average ChIP-seq signal of H3K27ac at the MYCN peak center. (**G**) Signal tracks show decreases in MYCN, H3K27ac, and H3K4me3 ChIP-seq signals at the promoter of *RPL8* after the depletion of MYCN (cyan box). (**H**) Signal tracks show increases in H3K27ac ChIP-seq signals at the promoter (cyan box) and enhancers (pink boxes) of *GAP43* after the depletion of *MYCN*. The data underlying the graphs in the figure are shown in S1 Data. IPA, Ingenuity Pathway Analysis.

Furthermore, we focused on MYCN-bound genes whose expression was modulated after changes in MYCN expression. After silencing *MYCN*, the integrative analysis of the MYCN ChIP-seq and RNA-seq merged data identified 601 genes whose expression decreased and thus was directly activated by MYCN (bound by 936 MYCN peaks), and 625 genes whose expression increased and thus was directly suppressed by MYCN (bound by 1,420 MYCN peaks) whose expression increased with *MYCN* silencing. Ingenuity Pathway Analysis (IPA) showed that MYCN-bound genes whose expression decreased after *MYCN* silencing were enriched in RNA posttranscriptional modification and protein synthesis (Fig 3A), whereas the MYCN-bound genes that were up-regulated after *MYCN* silencing were positively enriched in neuronal differentiation (Fig 3B).

Since we discovered that MYCN binds to the promoters of MYCN-activated canonical MYC target genes and binds to the enhancers of MYCN-repressed neuronal genes (Fig 1I), we next focused on how the silencing of *MYCN* affects the epigenetic modifications at MYCN binding sites within these promoters and enhancers. By focusing on the promoters of MYCN-bound down-regulated genes due to *MYCN* silencing, we found a decrease in the average ChIP-seq signals of active promoter marks H3K27ac at the MYCN peak center, while the decrease in the average H3K4me3 signals was not significant (Fig 3C). When focused on the promoters of MYCN-bound, up-regulated genes after *MYCN* silencing, we found an increase of the average ChIP-seq signal of H3K27ac at the MYCN peak center (Fig 3D). By focusing on the enhancers of MYCN-bound genes after *MYCN* silencing, there was an increase in the average H3K27ac ChIP-seq signal at the MYCN peak center for up-regulated genes (Fig 3E) but a decrease in the H3K27ac signal for down-regulated genes (Fig 3F). For example, signal tracks showed decreases of MYCN, H3K27ac, and H3K4me3 ChIP-seq signals at the promoter of *RPL8* (ribosomal protein L8) after the depletion of MYCN (Fig 3G), whereas signal tracks for the neuronal differentiation gene, *GAP43*, showed decreases of MYCN ChIP-seq signals and increases of H3K27ac ChIP-seq signals at both the promoter and enhancers after the depletion of MYCN (Fig 3H). These results suggest that MYCN directly activates canonical MYC target genes through increasing promoter activity, whereas MYCN directly represses neuronal differentiation genes through repressing enhancer activity.

Subsequently, we investigated the distribution of MYCN peaks after MYCN knockdown. ChIP-seq results revealed that silencing *MYCN* reduced the number of MYCN peaks from 19,707 to 6,012. Analyzing the distribution of MYCN peaks among the remaining 6,012 peaks following *MYCN* silencing in IMR32 cells through *k*-means clustering (S3C Fig) showed a significant decrease in the percentage of MYCN peaks within enhancers and a notable increase in the percentage of MYCN peaks within promoters, compared to the MYCN peak distribution in control cells (S3D Fig). This finding aligns with the observation that elevated levels of MYC oncoproteins tend to bind to distal regulatory regions [31–33].

### Interactome assay to identify MYCN novel cofactors

**Fig 4:**
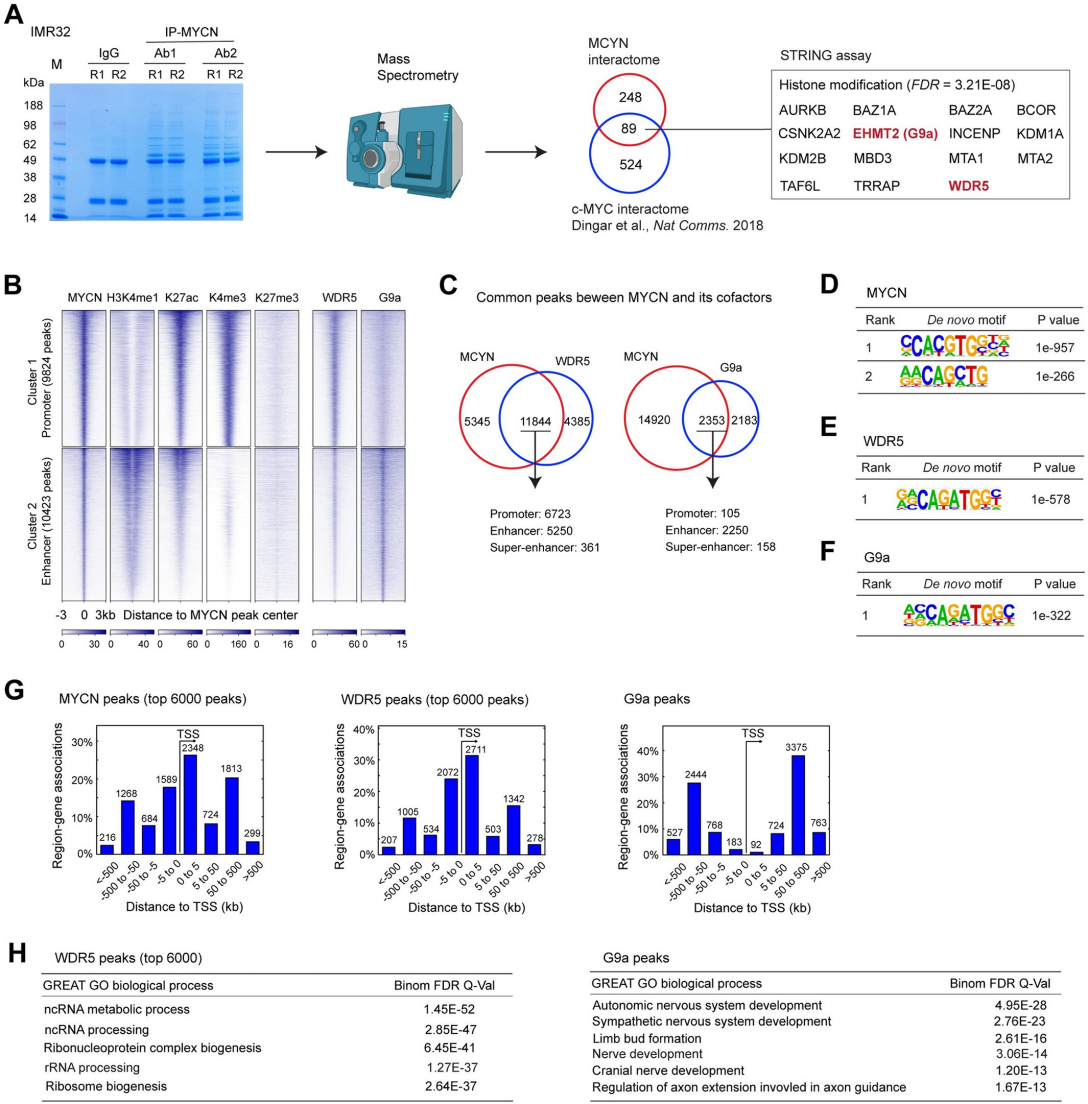
Genome-wide colocalization of MYCN and its cofactors. (**A**) Protein bands identified in the MYCN pulldown products in the protein gel (stained by Coomassie blue) are used for in-gel digestion and mass spectrometry sequencing (left panel). Discovered MYCN protein partners are compared with published c-MYC interactors (middle panel). GO analysis of the common protein interactors using STRING tool identifies protein enriched in histone modification (right panel). (**B**) *K*-Means clustering of MYCN and histone marks ChIP-seq and MYCN cofactors ChIP-seq around MYCN binding sites of NB cell line IMR32 (±3 kb) shows the overlapping binding sites between MYCN and its cofactors. (**C**) ChIPPeakAnno analysis shows the number of common and unique peaks between MYCN and its cofactors. (**D**–**F**) HOMER motif analysis shows that both canonical and non-canonical E-boxes are enriched in MYCN peaks, while non-canonical E-boxes are enriched in WDR5 and G9a peaks. (**G**) GREAT peak distribution analysis shows that around 50% of WDR5 peaks are within 5 kb from the TSS (left panel), while less than 5% of G9a peaks are within 5 kb from the TSS (right panel). (**H**) GREAT GO analysis indicates that WDR5-bound top ranked peaks associated genes are enriched in RNA processing and ribosome biogenesis (top panel), while G9a-bound peaks associated genes are enriched in nervous system development (bottom panel). The data underlying the graphs in the figure are shown in S1 Data. GO, Gene Ontology; GREAT, genomic regions enrichment of annotation tool; NB, neuroblastoma; TSS, the transcription start site.

TFs recruit cofactors to remodel the chromatin and/or modify histones to regulate gene transcription. To identify cofactors that mediate the transcriptional activity of MYCN on activating canonical MYC target genes and repressing differentiation genes, we performed co-immunoprecipitation (co-IP) coupled with mass-spectrometry assessments using 2 different MYCN antibodies to identify protein interactors of endogenous MYCN in IMR32 cells (Figs 4A and S4A). Each MYCN antibody pulled down around 400 protein partners, of which 337 were overlapped (S4 Table). The analysis confirmed multiple known MYCN and MYC partners, such as MAX, TRRAP, topoisomerases IIA and IIB (TOP2A and TOP2B) [35–37]. IPA of MYCN protein partners showed that 224 of the 337 MYCN protein partners (62%) are nuclear proteins (S4B Fig and S4 Table). The DAVID tool [38] was used to annotate MYCN protein partners within the nucleus and showed that these proteins were enriched in different categories such as chromosome organization, chromatin organization, intracellular ribonucleoprotein complex, DNA replication, and mRNA splicing (S4C Fig). Importantly, in support of our discovery, when compared to the recently identified, high-confidence interactors for c-MYC (S4 Table) in Hela cells which used a proximity-dependent biotinylation technique (BioID) [36], we found that 89 of the 337 MYCN interactors also were interactors of c-MYC (Fig 4A and S4 Table).

We focused on MYCN interactors with histone modifying enzymatic activity or those considered promising targets for anti-cancer therapies. GO analysis of the 89 proteins that interact with both MYCN and c-MYC using the STRING protein–protein interaction tool [39] identified 15 proteins that are significantly enriched in histone modifications (Fig 4A). Among these proteins, we focused on WDR5 (a component of histone methylation complex) and G9a (histone methyltransferase, also known as EHMT2). WDR5 is a coactivator of c-MYC and has been tested for anti-c-MYC therapies [40,41], while G9a is a corepressor of c-MYC [42]. WDR5 and G9a were found to be essential in many types of cancers including NB [14,43]. Moreover, WDR5 was found to facilitate MYCN recruitment to DNA [44]. However, assessments of global interactions of WDR5 and G9a in NB have not been investigated. We performed MYCN co-IP and western blot analysis in IMR32 cells and confirmed that MYCN could pull down both WDR5 and G9a (S4D Fig). Consistent with our discovery, a recent MYCN interactome assay performed in HEK293 cells with overexpressed MYCN (S4 Table) [37] also revealed that WDR5 and G9a interact with MYCN.

### Genome-wide colocalization of MYCN and its cofactors

To investigate the genome-wide interactions of MYCN and the coactivator WDR5 or corepressor G9a, we performed ChIP-seq analysis of MYCN, WDR5, and G9a in IMR32 cells. MYCN peaks were segmented based on their colocalizations with specific histone marks, WDR5, and G9a through *k*-means clustering. We found that WDR5 bound to both promoters and enhancers, although with a stronger signal intensity at the promoter regions (Fig 4B). In contrast, G9a predominantly bound to the enhancers (Fig 4B). ChIPPeakAnno analysis showed that 73% WDR5 and 52% G9a binding sites overlapped with MYCN binding sites (Fig 4C). Consistent with the heatmap shown in Fig 4B, ChIPPeakAnno analysis indicated that among the 11,844 MYCN and WDR5 overlapped peaks, 6,723 (56.8%) are within promoters, 5,250 (44.3%) are within enhancers, and 361 (3.0%) are within super-enhancers. For the 2,352 peaks where MYCN and G9a overlap, 105 (4.5%) are within promoters, 2,250 (95.6%) are within enhancers, and 158 (6.7%) are within super-enhancers (Fig 4C). Notably, a small number of peaks were assigned to both promoters and enhancers, or both enhancers and super-enhancers. HOMER motif scan showed that the top 2 MYCN binding motifs are canonical and non-canonical E-boxes, while the non-canonical E-box was found to be enriched in WDR5 and G9a binding sites (Fig 4D–4F). Consistent with the *k*-means clustering analysis, GREAT peak distribution analysis showed that few G9a binding sites were within 5 kb of TSS (<5%), while 55% of WDR5 binding sites were within 5 kb of TSS (Fig 4G). The genome-wide colocalization of MYCN with either WDR5 or G9a suggested that each of these cofactors cooperates with MYCN to regulate a subset of MYCN target genes. GREAT GO analyses showed that MYCN WDR5 ChIP-seq peak-associated genes were enriched in RNA processing and ribosome biogenesis (Fig 4H, left panel), suggesting its potential role as an MYCN coactivator. On the other hand, G9a binding site-associated genes were enriched in nervous system development (Fig 4H, right panel), suggesting its potential role as an MYCN corepressor. These results suggest that MYCN cooperates with WDR5 to regulate canonical MYC target genes while MYCN cooperates with G9a to regulate neuronal differentiation genes.

### *MYCN* silencing alters the genomic DNA binding of its cofactors

**Fig 5:**
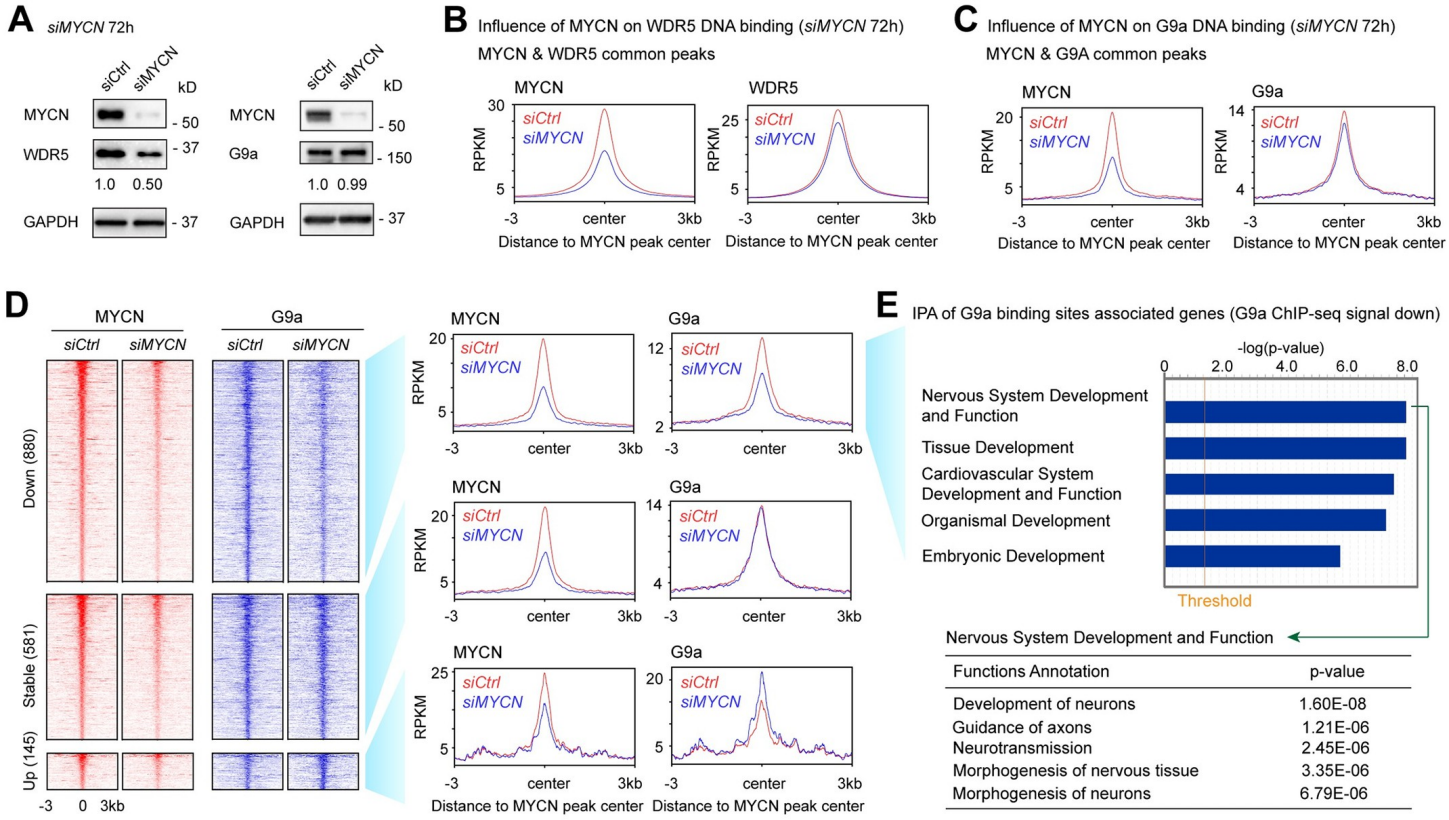
Silencing of *MYCN* selectively alters genomic DNA binding of its cofactors. (**A**) Western blot and densitometric analyses show the effect of 72 h *MYCN* knockdown in IMR32 cells on the expression of its cofactors at the protein levels. (**B**) Metagene plots show that the knockdown of *MYCN* results in a decrease of average MYCN and WDR5 ChIP-seq signal at the MYCN peak center of the MYCN and WDR5 overlapped binding sites. (**C**) Metagene plots show that the knockdown of *MYCN* results in a decrease in average MYCN and G9a signal at the MYCN peak center of MYCN and G9a overlapped binding sites. (**D**) ChIP-seq heatmaps (left) and profiles (right) of MYCN and G9a in control and *MYCN* knockdown IMR32 cells around MYCN binding sites (±3 kb) of MYCN and G9a overlapped peaks. (**E**) IPA of the G9a ChIP-seq data shows that the G9a binding sites with decreased ChIP-seq signal (>1.2-fold decrease of G9a signal after the silencing of *MYCN*) associated genes are enriched in nervous system development. The data underlying the graphs in the figure are shown in S1 Data. IPA, Ingenuity Pathway Analysis.

To identify whether MYCN recruits its cofactors to its binding sites, we performed ChIP-seq experiments using MYCN, WDR5, and G9a antibodies in *siCtrl* and *siMYCN* transfected IMR32 cells. *MYCN* silencing did not alter the steady-state protein levels of G9a but did cause a 50% decrease in WDR5 based on densitometric analysis (Fig 5A). ChIP-seq results showed that the knockdown of *MYCN* in IMR32 cells caused a 15% decrease in WDR5 ChIP-seq peak numbers (18,263 to 15,612 peaks) (S5 Table), which was possibly caused by the decrease of WDR5 protein. By focusing on MYCN and WDR5 overlapped peaks, metagene plots showed a 50% decrease in the average MYCN ChIP-seq signals and a 5% decrease in the average WDR5 ChIP-seq signals at the summit of MYCN peak centers (Fig 5B) after silencing of *MYCN*. For example, signal tracks for the ribosome gene *RPL8* promoter and the cell adhesion molecule coding gene *PVR* promoter showed decreases in WDR5 binding after *MYCN* silencing (S5A Fig). However, it is worth noting that silencing *MYCN* resulted in a 50% decrease of WDR5 at the protein level but was accompanied by only a slight decrease in WDR5 ChIP-seq peak numbers and average ChIP-seq signal intensity, suggesting that the interaction between MYCN and DNA is not required for WDR5 to bind to DNA.

To investigate the WDR5-independent MYCN target genes, we compared the list of genes regulated by MYCN (silencing *MYCN* for 48 h) and the list of genes regulated by WDR5 (silencing *WDR5* for 48 h) by analyzing the RNA-seq data (threshold, >1.5-fold and <−1.5-fold, q < 0.05) in IMR32 cells. We found that among the 1,908 MYCN target genes, 83% (1,598/1,908 genes) were regulated by MYCN but not by WDR5. Among the WDR5 target genes, 66% (610/920 genes) were regulated by WDR5 but not by MYCN (S6 Table). IPA showed that genes commonly regulated by MYCN and WDR5, or uniquely regulated by WDR5, are involved in regulating protein translation, while genes only regulated by MYCN are enriched in regulating nervous system development and other pathways (S7 Table). These results indicate that MYCN regulates broad transcriptional programs, which overlap with WDR5 to regulate transcriptional programs associated with protein translation.

When focused on G9a, we found that MYCN silencing resulted in a 64% decrease in G9a ChIP-seq peak numbers (6,723 to 2,428 peaks) (S8 Table). Metagene plots of MYCN and G9a overlapping peaks show a 5% decrease in the average G9a ChIP-seq signals at the summit of MYCN peak centers after silencing MYCN (Fig 5C). For example, signal tracks for the *KCNK3* gene showed that *MYCN* knockdown decreased MYCN and G9a binding signals within the *KCNK3* intron (S5B Fig). *MYCN* silencing resulted in a subtle decrease in the average G9a ChIP-seq signals (Fig 5C) but dramatically reduced G9a ChIP-seq peak numbers (S8 Table). Thus, we further analyzed the MYCN and G9a overlapping peaks by dissecting them into 3 clusters. The first cluster was classified as down-regulated (down) peaks, which included G9a peaks with at least >1.2-fold decrease of G9a ChIP-seq signal after *MYCN* silencing (Fig 5D); the second cluster was classified as not-altered (stable) peaks, which included G9a peaks with <1.1-fold changes of G9a ChIP-seq signal after the silencing of *MYCN* (Fig 5D); whereas the third cluster was classified as increased (up) peaks, which included G9a peaks with >1.2-fold increase of G9a ChIP-seq signal after the silencing of *MYCN* (Fig 5D). More G9a binding peaks showed decreases in their ChIP-seq signal than the ones that were stable or had increased signals in *MYCN* silenced cells (Fig 5D). This is consistent with the observation of a reduced number of G9a ChIP-seq peaks after the silencing of *MYCN* (S8 Table). IPA of the G9a peaks associated genes with a decreased or stable ChIP-seq signal revealed that these genes were enriched in nervous system development (Figs 5E and S5C). Furthermore, the genes associated with G9a binding sites whose ChIP-seq signals increased were enriched in the development of other tissues such as hair and skin development (S5D Fig). Our results indicated that MYCN selectively recruits G9a to MYCN binding sites that are associated with neuronal genes.

### MYCN cofactors facilitate MYCN binding to DNA

**Fig 6:**
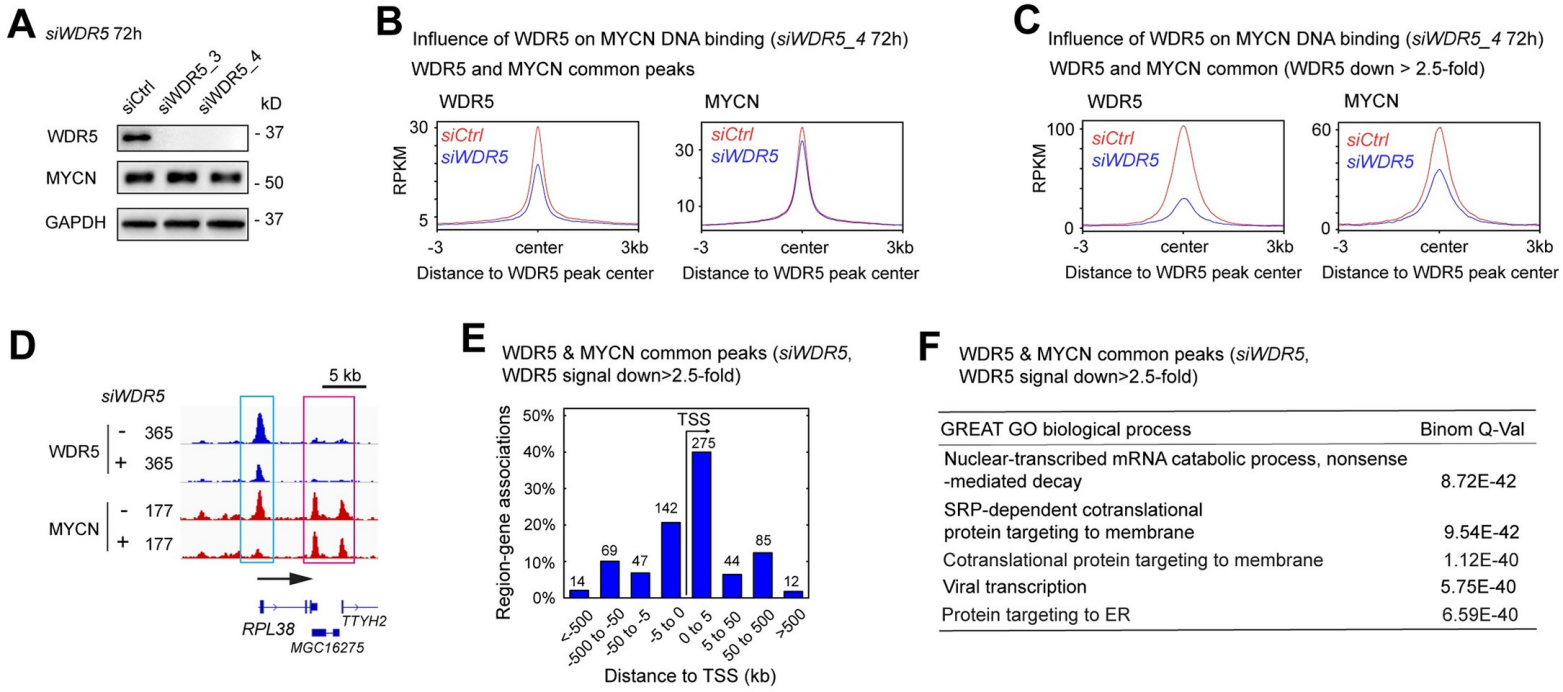
MYCN cofactors assist MYCN to bind to DNA. (**A**) Western blot analysis shows that *WDR5* knockdown using siRNAs for 72 h decreases in WDR5 protein with no effect on MYCN expression. (**B**) and (**C**) Metagene plots show that *WDR5* knockdown decreases average WDR5 and MYCN ChIP-seq signals at the WDR5 peak center. The decreases in average MYCN signal are more dramatic when focused on WDR5 and MYCN overlapped binding sites with >2.5-fold decrease of WDR5 ChIP-seq signal intensity. (**D**) Signal tracks show that *WDR5* knockdown decreases MYCN signal at the promoter of the *RPL38* gene (cyan box) but not at the *TTYH2* gene locus (pink box). (**E**) When focused on WDR5 and MYCN overlapping peaks with >2.5-fold decrease of WDR5 ChIP-seq signals, GREAT peak distribution analysis shows that around 60% of WDR5 and MYCN overlapped peaks are within 5 kb away from the TSS, and (**F**) GREAT GO biological process analysis shows that these WDR5 and MYCN overlapped peaks associated genes are enriched in RNA processing and protein synthesis. The data underlying the graphs in the figure are shown in S1 Data. GO, Gene Ontology; GREAT, genomic regions enrichment of annotation tool; TSS, the transcription start site.

Recent studies showed that target gene recognition by c-MYC is not solely dependent on interactions with MAX, but also depends on other proteins including WDR5 [40,41]. Mutations in c-MYC that disrupt the WDR5 interaction result in a significant decrease in the binding of c-MYC to its target genes [40]. To investigate whether WDR5 is required for MYCN to bind to DNA, we performed ChIP-seq analysis before and after the silencing of *WDR5*. Western blot results showed that the silencing of *WDR5* did not alter the expression of MYCN protein (Fig 6A).

When focused on WDR5 and MYCN overlapping binding sites, metagene plots showed that the silencing of *WDR5* resulted in a decrease in the average ChIP-seq signal of MYCN (Fig 6B). We next investigated the genomic loci where WDR5 assisted MYCN binding by focusing on MYCN-bound promoters and enhancers as defined by the histone marks (Fig 1E). Metagene plots showed that the knockdown of *WDR5* resulted in an approximately 20% decrease in average MYCN ChIP-seq signal at the summit of MYCN-bound promoters and a 10% decrease in MYCN signal at the summit of MYCN-bound enhancers (S6A and S6B Fig), suggesting that WDR5 has a greater effect on MYCN binding to promoters compared to enhancers. Of note, the knockdown of *WDR5* resulted in >90% depletion of WDR5 protein levels detected by western blot (Fig 6A), but it only reduced the number of WDR5 ChIP-seq peaks by 52% (15,448 to 7,404 peaks) (S9 Table), and there was only a 40% decrease in WDR5 ChIP-seq signal intensity at the summit of the WDR5 peak center (Fig 6B, left panel). This suggests that the remaining WDR5 after *WDR5* knockdown binds to certain genomic loci with high WDR5 binding affinity. To investigate how the depletion of WDR5 influenced MYCN-DNA interaction on these binding sites, we focused on the WDR5 and MYCN overlapping binding sites with >2.5-fold decrease in WDR5 ChIP-seq signals after *WDR5* silencing (Fig 6C, left panel). Metagene plots showed an obvious decrease (50%) in MYCN ChIP-seq signals at these genomic loci (Fig 6C, right panel), indicating that WDR5 is required for MYCN to bind DNA on these genomic loci. For example, signal tracks showed that the silencing of *WDR5* resulted in a >70% decrease of both WDR5 and MYCN ChIP-seq signals at the promoter of ribosome biogenesis gene *RPL38* (Fig 6D, cyan box). However, when focusing on the WDR5 and MYCN overlapped binding sites with a <1.2-fold decrease of WDR5 ChIP-seq signals after the silencing of *WDR5*, the metagene plots showed only a 10% decrease in MYCN ChIP-seq signals at these genomic loci (S6C Fig). For example, signal tracks showed that the silencing of *WDR5* did not alter the WDR5 ChIP-seq signal or the MYCN ChIP-seq signals at the promoter of the *NAT9* gene and *TMEM104* genes (S6D Fig).

Furthermore, we performed ChIP-re-ChIP experiments by immunoprecipitating chromatin with an anti-MYCN antibody followed by an anti-WDR5 antibody. ChIP-PCR results showed that both the first ChIP with the anti-MYCN antibody and the second re-ChIP with the anti-WDR5 antibody pulled down DNA fragments within the *RPL38* promoter region (S6E Fig). This demonstrates that these 2 proteins are indeed in the same complex that binds to DNA.

Taken together, these results support that WDR5-DNA binding is required for MYCN to bind DNA on these genomic loci. Despite these observations, we cannot rule out that the decreased cell proliferation and cell-cycle progression after *WDR5* depletion might reduce MYCN binding on certain genomic loci.

To investigate which genes were cooperatively bound by WDR5 and MYCN, we focused on the WDR5 and MYCN overlapping binding sites with a >2.5-fold decrease in WDR5 ChIP-seq signals after the silencing of *WDR5*. GREAT peak distribution analysis showed that 60% of these peaks were within 5 kb from the TSS, while the remaining were 5 kb away from the TSS (Fig 6E). This indicated that WDR5 mainly assisted MYCN binding to promoters. GREAT GO analysis indicated that these WDR5 and MYCN overlapped peak-associated genes are significantly enriched in protein synthesis, RNA processing, and ribosome biogenesis, but not in nervous system development (Fig 6F and S10 Table). Taken together, these findings indicate that WDR5 mainly assists MYCN to bind to the promoters that are associated with MYCN-activated canonical MYC targets.

### The depletion of MYCN cofactors antagonizes MYCN-mediated gene expression changes

Our study indicated that WDR5 and G9a interact with MYCN and that these proteins colocalize to the promoters or enhancers of genes they regulate (Fig 4B). To investigate whether the presence of WDR5 or G9a is necessary to regulate MYCN target genes, we silenced *WDR5*, *G9a*, or *MYCN* using siRNAs for 48 h in IMR32 cells and performed RNA-seq analysis. Western blot results showed that the silencing of *WDR5* or *G9a* had no effect on MYCN expression at this time point (S7A Fig). After *MYCN* silencing for 48 h in IMR32 cells, GSEA results showed significant negative enrichment of canonical *MYC* target genes involved in ribosome biogenesis, RNA processing, ribosome formation, and cytoplasmic translation (S7B Fig), with significant positive enrichment of neuronal genes involved in axon development, neuron differentiation, glutamatergic synapse, and neuron projection guidance (S7C Fig). To compare genes regulated by MYCN, WDR5, and G9a in IMR32 cells after 48 h *MYCN* silencing, we generated an “MYCN-activated canonical MYC targets” gene sets by defining genes that were significantly down-regulated after the silencing of *MYCN* in the gene sets of ribosome biogenesis, RNA processing, ribosome formation, and cytoplasmic translation (S11 Table). In parallel, we generated an “MYCN-repressed neuronal genes” gene sets by defining those genes that were significantly up-regulated after the silencing of *MYCN* in the gene sets of axon development, neuron differentiation, glutamatergic synapse, positive regulation of synaptic transmission, and neuron projection guidance (S11 Table).

**Fig 7:**
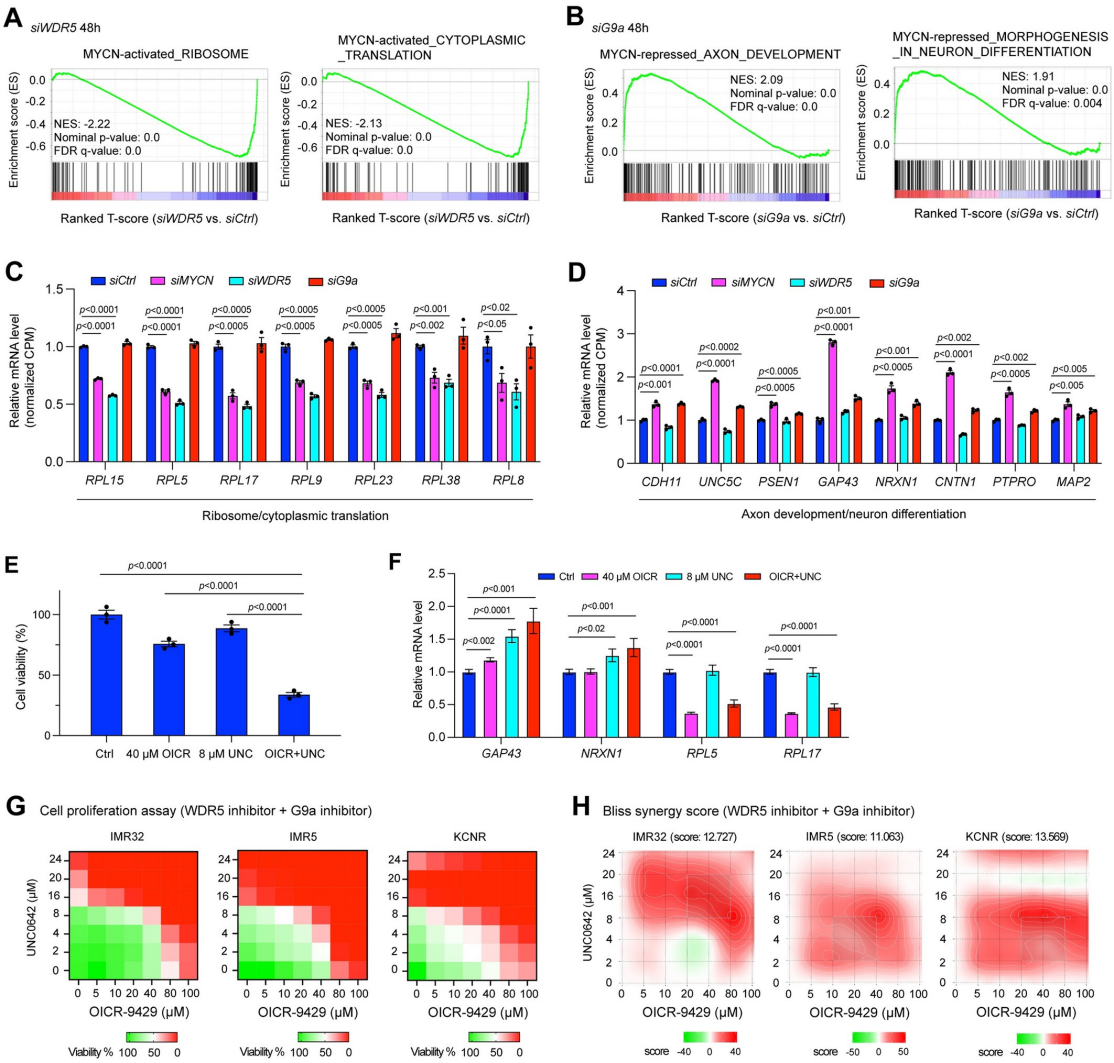
The depletion of MYCN cofactors antagonizes MYCN-mediated gene transcription regulation, which makes them potential therapeutic targets. (**A**) GSEA shows that the silencing of *WDR5* results in a significant negative enrichment of genes involved in ribosome formation and protein synthesis that are activated by MYCN. (**B**) GSEA shows that the silencing of *G9a* results in a significant positive enrichment of genes involved in axon development and neuron differentiation that are repressed by MYCN. (**C**) The silencing of either *MYCN* or *WDR5* but not *G9a* results in a significant down-regulation of genes involved in ribosome formation and protein translation based on the RNA-seq results. The *p*-value indicated is calculated in one-way ANOVA. (**D**) The silencing of either *MYCN* or *G9a* but not *WDR5* results in a significant up-regulation of genes involved in axon development and neuron differentiation based on the RNA-seq results. The *p*-value indicated is calculated in one-way ANOVA. (**E**) CellTiter-Glo assay shows the drug effect of the OICR-9429 (OICR, 40 μm) + UNC0642 (UNC, 8 μm) treatment on IMR32 cell viability at 72 h. (**F**) Realtime PCR shows that the inhibition of both WDR5 and G9a results in a significant down-regulation of ribosomal genes and up-regulation of neuronal genes. (**G**) Heatmaps show the percentage of cell viability after different doses of WDR5 inhibitor OICR-9429 and G9a inhibitor UNC0642 treatment in *MYCN*-amplified NB cell lines. Cells are treated with the drugs for 72 h and cell viability is measured by CellTiter-Glo Cell Viability Assay. (**H**) SynergyFinder online tool is used for bliss synergistic analysis to evaluate the synergistic effect of the combination treatment in *MYCN*-amplified NB cell lines shown in (**G**). The data underlying the graphs in the figure are shown in S1 Data. CPM, counts per million; GSEA, gene set enrichment analysis; NB, neuroblastoma.

The silencing of *WDR5* resulted in a significant negative enrichment of “MYCN-activated canonical MYC targets” gene set such as genes involved in ribosome formation and cytoplasmic translation (Fig 7A). GSEA results did not exhibit significant positive enrichment of neuronal genes after the silencing of *WDR5*.

G9a predominantly binds to the enhancers (Fig 4B) and G9a-bound peak-associated genes were enriched in nervous system development (Fig 4H). GSEA results showed that the silencing of *G9a* resulted in a significant positive enrichment of the “MYCN-repressed neuronal genes” gene set such as genes involved in axon development, neuron differentiation, glutamatergic synapse, and neuron projection guidance (Figs 7B and S7D), while no significant negative enrichment of canonical MYC target genes was observed after the silencing of *G9a*.

Representative genes commonly down-regulated after the silencing of *MYCN* or *WDR5* that belong to the gene sets of “MYCN-activated canonical MYC targets” were shown in Fig 7C. The same group of genes was not down-regulated after *G9a* silencing (Fig 7C). Representative genes commonly up-regulated after the silencing of *MYCN* or *G9a* that belong to “MYCN-repressed neuronal genes” gene set were shown in Fig 7D. The same group of genes was not up-regulated after the silencing of *WDR5* (Fig 7D). These results indicated that *WDR5* silencing antagonizes MYCN-mediated activation of canonical MYC target genes, while *G9a* silencing antagonizes MYCN-mediated repression of neuronal genes.

### Targeting both MYCN coactivator and corepressor simultaneously

It has been shown that the treatment of NB cells with either a WDR5 inhibitor alone or a G9a inhibitor alone inhibits NB growth [14,45–47]. Since WDR5 cooperates with MYCN to activate a canonical MYC target gene program while G9a cooperates with MYCN to repress a neuronal differentiation gene program, we hypothesized that a more efficient strategy to drug MYCN oncogenic activities would be to target both these cofactors simultaneously.

First, we evaluated whether the genetic inhibition of *WDR5* or *G9a* affects NB cell proliferation. DepMap (https://depmap.org/portal/) CRISPR library screen data analysis showed that *WDR5* was essential in all the NB cell lines while half the NB cell lines were partially dependent on *G9a* to survive or proliferate based on the CRISPR dependence score (S7E Fig). Of note, the *WDR5* or *G9a* dependency was not dependent on *MYCN* amplification status (S7E Fig), which is possible due to the high expression of *c-Myc* in *MYCN* non-amplified NB cell lines [48], while WDR5 and G9a have been shown to mediate c-MYC function in the other types of cancers [40,42]. Consistent with the DepMap CRISPR library screen results, we found that the genetic silencing of *WDR5* or *G9a* using siRNAs in IMR32 cells decreased in NB cell proliferation (S7F Fig), supporting the dependency of NB cells on each of these cofactors.

Next, as a proof of concept, we evaluated the ability of a small molecule inhibitor selective for WDR5 and one for G9a on MYCN target genes’ expression and NB cell proliferation. OICR-9429 is a WDR5 WIN site inhibitor that displaces WDR5 from chromatin [49]. UNC0642 is a G9a catalytic inhibitor in which the anti-proliferative response to UNC0642 correlates with MYC sensitivity and gene signatures in breast cancer cell lines [42]. A representative IMR32 cell proliferation assay showed that even though using a single dose of OICR-9429 or UNC0642 that only slightly reduced cell viability (10% to 25%), the combination of these 2 drugs dramatically reduced cell viability (>60%) after 72 h drug treatment (Fig 7E), which indicated a potential synergistic effect. WDR5 inhibition alone significantly decreased the expression of ribosome genes *RPL5* and *RPL17* while G9a inhibition alone increased the expression of neuronal genes *GAP43* and *NRXN1*, whereas the combination up-regulated *GAP43* and *NRXN1* and repressed *RPL5* and *RPL17* mRNA levels (Fig 7F). Further cell proliferation assays showed that the treatment of NB cells with increased dosages of either OICR-9429 or UNC0642 alone reduced the number of viable NB cells (Fig 7G), while the combination of OICR-9429 and UNC0642 more dramatically reduced NB cell proliferation (Fig 7E and 7G). Next, we utilized the SynergyFinder online tool (https://synergyfinder.fimm.fi/) for bliss synergistic analysis. A synergy score ranging from −10 to 10 indicates an additive interaction between the 2 drugs, while a score greater than 10 suggests a synergistic interaction. We observed that the combination of OICR-9429 and UNC0642 synergistically reduced NB cell proliferation, as evidenced by an average bliss synergy score exceeding 10 across a range of doses in IMR32, IMR5, and KCNR cells (Fig 7H). These results indicate that inhibiting both MYCN coactivators and corepressors is necessary to repress both the active and repressive activity of MYCN, thereby resulting in a synergistic effect on suppressing NB cell proliferation.

## Discussion

**Fig 8:**
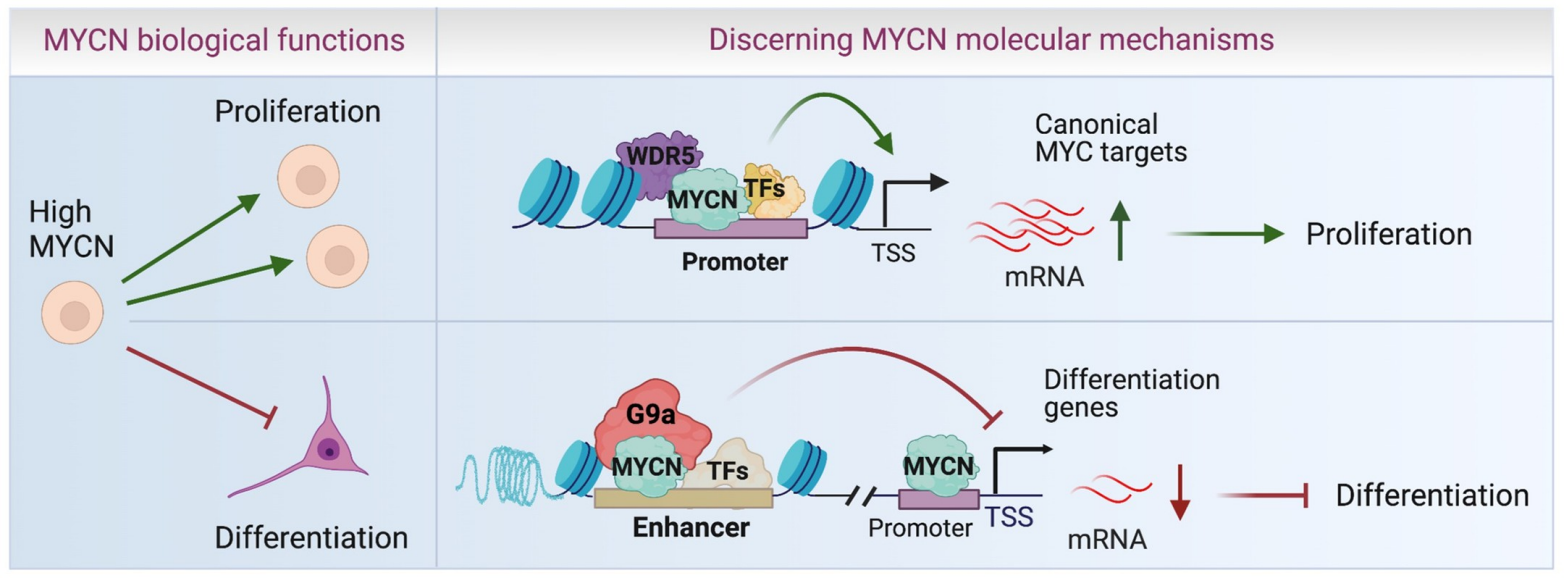
Schematic diagram of MYCN action. WDR5 assists MYCN to bind promoters and up-regulate canonical MYC target genes to stimulate cell proliferation, whereas MYCN recruits G9a to enhancers to down-regulate neuronal differentiation genes and inhibit cell differentiation.

MYCN is a common oncogene in many types of cancers, yet how MYCN regulates global gene expression has not been well-characterized. Through genome-wide and proteomic approaches, we have identified critical interactions between MYCN and the transcriptional coactivator WDR5 and corepressor G9a and mapped their interactions at a genome level in NB cells. We find that WDR5 facilitates MYCN binding to genomic DNA to activate canonical MYC target genes involved in protein synthesis, which occurs mainly via promoter binding. In contrast, MYCN recruits corepressor G9a to bind enhancers and this functions to directly repress neuronal differentiation gene programs (Fig 8). MYCN requires these cooperative interactions to mediate its oncogenic transcriptional program by using a coactivator to stimulate growth-supporting pathways and a corepressor to inhibit the growth-inhibiting pathways that would naturally occur as cells differentiate (Fig 8). In this way, MYCN orchestrates global gene expression and governs the malignant NB cell identify. Targeting the cofactors that mediate these 2 pathways antagonizes the dysregulated MYCN activity and more effectively suppresses NB tumor cell growth.

Although it was known that MYCN activates canonical MYC targets involved in ribosome biogenesis, protein synthesis, and RNA processing [1,9,10], here we find at a genome-wide level that this is mainly through MYCN binding to promoters. MYCN has been reported to repress neuronal differentiation genes [11], but whether this was direct or indirect had only been assessed on a small number of genes. Here, we demonstrate that in NB cells, MYCN binds to both the promoters and the enhancers of neuronal differentiation genes, and there is a significant up-regulation of these genes upon depletion of *MYCN* from these binding sites. Previous studies showed that MYCN cooperates with MIZ1 or SP1 to repress gene transcription through binding to the MIZ1 or SP1 binding site within the promoters [50,51]. For the first time, we find that in addition to promoter binding, MYCN utilizes enhancer binding to repress gene expression in NB cells, as the depletion of MYCN from these enhancers is associated with activated histone modifications as shown by the increase in H3K27ac ChIP-seq signals. This observation is consistent with the well-known concept that enhancers control cell type-specific gene expression [52]. Moreover, our discovery of the repression of neuronal differentiation genes by MYCN is consistent with the observation that the silencing of *MYCN* in NB cells results in an increase in neuronal differentiation. In another MYCN driven tumor, RMS, MYCN binds to enhancers and directly represses muscle differentiation genes, suggesting a general mechanism by which enhancer-bound MYCN represses differentiation genes. Our model in which MYCN selectively activates canonical MYC targets through binding promoters and represses cell lineage-specific differentiation genes through binding enhancers more fully rationalizes the different oncogenic cellular effects driven by MYCN in different types of cancer cells.

Our study dissects the molecular mechanisms by which MYCN activates canonical MYC target genes and represses neuronal genes. The coactivator WDR5 has been shown to interact with c-Myc and MYCN [14,40]. WDR5 was found to recruit c-MYC to chromatin and regulate the expression of protein synthesis genes [40,41]. Consistent with this, we find that WDR5 facilitates MYCN genome binding to activate canonical MYC target genes involved in ribosome biogenesis and protein synthesis. We demonstrate that many neuronal differentiation genes are repressed by MYCN and MYCN binds to the enhancers of these genes. MYCN has been shown to alter bivalent epigenetic marks (H3K4me3 and H3K27me3) by recruiting PRC2 complex to transcriptionally repress expression of CLU gene [17]. Components of the PRC2 complex have not been identified in our MYCN interactome assay, and in genome-wide assessments, MYCN did not colocalize with H3K27me3 on the genome (Fig 1E), suggesting that the direct repression of most of the neuronal genes by MYCN is not via PRC2 recruitment. It is possible that MYCN regulates some of these neuronal genes indirectly through PRC2 since it is known that MYCN activates EZH2 expression [53]. A recent study indicated that G9a cooperates with c-MYC to repress gene transcription [42]. G9a is responsible for H3K9 dimethylation that is known to be associated with transcriptional repression [54,55]. In our study, we find that MYCN recruits G9a to the enhancers of neuronal differentiation genes, which possibly catalyzes H3K9me2, establishing a repressive chromatin environment at MYCN binding sites to decrease enhancer activity. A limitation of our study is that we have not been able to successfully perform an H3K9me2 ChIP-seq to determine how the recruitment of G9a to MYCN binding sites affects H3K9me2 status despite the use of most commercially available H3K9me2 antibodies. Nevertheless, our findings that MYCN colocalizes with G9a on enhancers associated with neuronal differentiation genes, and the finding that the depletion of *G9a* antagonizes MYCN-mediated repression of neuronal genes, support a model in which G9a functions as an MYCN corepressor to suppress cell type-specific differentiation genes. As others have proposed [56], it is highly likely MYCN engages in several protein complexes rather than forming a single defined complex in regulating gene transcription. We find that MYCN mainly cooperates with WDR5 at the promoters while cooperating with G9a at the enhancers, suggesting that MYCN forms different protein complexes with WDR5 and G9a. In addition to G9a, our MYCN interactome assay identified other corepressors or chromatin remodeling complexes such as the NuRD complex, suggesting that these corepressors might also participate with MYCN to contribute to the suppression of certain neuronal differentiation genes.

In tumors driven by MYCN, therapeutic targeting of MYCN has been a long-sought goal, but has remained challenging due to its structure flexibility [5]. The intrinsic enzyme activity of cofactors offers the potential for developing strategies aimed at the indirect therapeutic targeting of MYCN. It has been shown that a WDR5 inhibitor was used to treat NB when WDR5 was found to be a coactivator of MYCN [14,45]. In our study, we demonstrate that WDR5 only mediates the active transcriptional activity of MYCN in activating protein synthesis genes and RNA processing genes. Thus, targeting WDR5 alone does not fully suppress the transcriptional activity of MYCN that contribute to uncontrolled growth. We find that G9a mediates the repressive transcriptional activity of MYCN in repressing neuronal differentiation genes in NB, and this led to a strategy that simultaneous inhibition of both WDR5 and G9a would more fully target MYCN oncogenic transcriptional activities. Indeed, the combination of WDR5 inhibitor and G9a inhibitor synergistically suppressed NB cell proliferation. This discovery highlights that targeting both coactivators and corepressors of an oncogenic TF simultaneously enables more precise therapy.

In summary, genome-wide mapping of MYCN binding and transcriptome analysis indicates that MYCN binds to promoters to activate canonical *MYC* target genes, whereas MYCN binds both enhancers and promoters to repress tissue-specific differentiation genes. Our results indicate that the oncogenic competence of MYCN is mediated by a combination of its coactivators including WDR5, and its corepressors including G9a. MYCN forms a dynamic complex system with different cofactors on different genomic loci to control the chromatin landscape, guide the expression of genes, and determine the cancer cell identity. Since WDR5 cooperates with MYCN to activate a canonical MYC target gene program that fuels cell proliferation while G9a cooperates with MYCN to repress a neuronal differentiation gene program that puts a brake on differentiation, the combined targeting of WDR5 and G9a simultaneously antagonizes these MYCN-mediated gene regulatory programs to synergistically suppress NB cell proliferation. The delineation of the mechanistic underpinnings of MYCN oncogenic activity in pediatric embryonal tumors provides a rationale to target both these cofactors simultaneously, which has important therapeutic implications for patients with *MYCN*-driven tumors.

## Materials and methods

### Cell culture

Human NB cell lines IMR32, SK-N-BE(2)C (BE(2)C), SMS-KCNR (KCNR), LAN5, and SHEP were obtained from the cell line bank of the Pediatric Oncology Branch of the National Cancer Institute and have been genetically verified. Human rhabdomyosarcoma cell line RH4 was from Dr. Javed Khan’s lab of the Genetic Branch of the National Cancer Institute. All the NB cell lines and rhabdomyosarcoma cell line were maintained in RPMI-1640 medium. All the cell culture medium was supplemented with 10% fetal calf serum (FBS), 100 μg/ml streptomycin, 100 U/ml penicillin, and 2 mM L-glutamine. Cells were grown at 37°C with 5% CO2. All cell lines were frequently assayed for *Mycoplasma* using MycoAlert Kit (Lonza) to ensure they were free of *Mycoplasma* contamination. The cell lines used were within 12 passages after thawing.

### Stable clones

HA tagged MYCN (HA-MYCN) construct was generously provided by Dr. Wei Gu’s lab [57]. MYCN coding region was PCR amplified from HA-MYCN construct and cloned into the doxycycline inducible pLVX-pTetOne-puro vector (Takara Bio) using In-Fusion HD kit (Takara Bio) following the manufacturer’s manual. SHEP cells were infected with lentiviral particles generated using the pLVX-TetOne-Puro-MYCN vector, followed by puromycin (0.65 μg/ml) selection. The transduced stable cell line was named as SHEPtetMYCN. MYCN expression in SHEPtetMYCN could be induced with 0.25 μg/ml Dox treatment.

### Transient transfection

siRNA control (AllStars Negative Control siRNA, Catalog No. 1027281) and siRNAs targeting different genes (Hs_MYCN_2, Catalog No. SI00076293; Hs_MYCN_4, Catalog No. SI00076307; Hs_G9a_3, Catalog No. SI00091203; Hs_WDR5_3, Catalog No. SI00118916; Hs_WDR5_4, Catalog No. SI00118923) were purchased from Qiagen or Santa Cruz Biotechnology. siRNAs were transiently transfected into NB cells using Nucleofector electroporation (Lonza): solution L and program C-005 for IMR32; solution V and program A-030 for the rest NB cell lines; solution R and program T-016 for RH4 cell line.

### Cell growth and neurite extension assay

To evaluate cell proliferation, NB cells were plated in 96-well plates and the growth kinetics were monitored in IncuCyte ZOOM or FLR (Essen BioScience) using the integrated confluence algorithm as a surrogate for cell number. Cell neurite length was measured using Essen IncuCyte ZOOM neurite analysis software.

### Monitoring of synergistic effects of drug combinations

The therapeutic effect of WDR5 inhibitor (WDR5i) OICR-9429 (Selleckchem, Catalog No. S7833) and G9a inhibitor (G9ai) UNC0642 (Selleckchem, Catlog No. S7230) in MYCN-Amp NB cell lines IMR32, KCNR, and IMR5 was determined in a checkerboard fashion. Cell lines were seeded in two 96-well plates and incubated overnight. Each combination dose had 2 replications. The next day, cell lines were treated with different dose combinations of OICR-9429 and UNC0642. Control cells were treated with DMSO. Each plate has its control cells. Cell viability was determined after 72 h using the CellTiter-Glo luminescent assay (Promega, catalog number G9242). Cell viability of DMSO-treated cells was set to 100%. Results were graphed with GraphPad Prism (RRID:SCR_002798). IncuCyte assay was used for testing the impact of synergistic effects of drug combinations on NB cell growth in realtime. Representative data from biological replicates were shown in this study. SynergyFinder (RRID:SCR_019318) online tool [58] was used to study the synergistic effect of the combination treatment of NB cells in vitro.

### Soft agar assay

To assess the effects of overexpression of *MYCN* in SHEP cells on anchorage independent cell growth, 1 × 10^4^ SHEPtetMYCN cells were cultured in 0.7% top agarose in media on a layer of 1.4% bottom agar/media to prevent the adhesion of cells to the culture plates. Medium was changed twice a week with or without 0.5 μg/ml Dox, and visible colonies were observed after 2 to 4 weeks of culture. The number of colonies was counted after crystal violet staining.

### Protein isolation, western blotting analysis, and co-immunoprecipitation

For assessment of protein levels, cells were lysed using RIPA buffer, and 10 μg of total protein was separated and electroblotted. Protein bands probed with diluted primary antibodies (S12 Table) were detected using a goat anti-rabbit or mouse IgG-HRP conjugated secondary antibody (Santa Cruz Biotechnology) and visualized using enhanced chemiluminescence (Amersham Biosciences).

To identify the interactome of the endogenous MYCN, co-IP was performed as previously described with slight modification [59]. IMR32 cells were solubilized for 30 min in cold lysis buffer (50 mM (pH 7.5) Tris-HCl, 137 mM NaCl, 1 mM DTT, 1 mM EDTA, 0.5% Triton X-100) supplemented with protease and phosphatase inhibitors (Halt protease and phosphatase inhibitor, Thermo), by shaking at 4°C. Two different MYCN antibodies (antibody 1, Santa Cruz, sc-53993; antibody 2, Abcam, ab16898) (4 μg) or normal IgG (4 μg) was incubated with 50 μl Dynabeads M-280 sheep anti-mouse IgG magnetic beads (Thermo Fisher Scientific Cat# 11201D, RRID:AB_2783640) in 200 μl wash buffer (50 mM (pH 7.5) Tris-HCl, 137 mM NaCl, 1 mM EDTA, 0.5% Triton X-100) overnight with rotation at 4°C. The clear cell lysate (20 mg) was incubated with the Magnetic Beads coupled with MYCN antibody 1, MYCN antibody 2, or IgG control in total 4.5 ml lysis buffer and agitated at 4°C for 4 h. Subsequently, the beads were washed 5 times with washing buffer. The co-IP products were eluted by incubating with 25 μl 1× LDS-PAGE sample buffer supplemented with 10% β-mercaptoethanol and boiling for 5 min. After staining with SimplyBlue Safe Stain reagents, the differentially pulled-down bands were sequenced using mass spectrometry (mass-spec) (NCI-Frederick protein analysis core facility). To validate the mass-spec result, co-IP and western blot were performed, and in this validation experiment, Benzonase (500 U/ml), Mg2+ (2 mM) will be added to the co-IP reaction as indicated. Benzonase is a nuclease that digests both DNA and RNA. Of note, for the mass-spec assay, we did not add Benzonase since we aim to identify most of MYCN interactors including those weak interactions that occur when the complex bind to nucleic acids. Primary antibodies of MYCN, G9a, and WDR5 (S12 Table) were used to detect the protein–protein interaction.

### RNA-seq

Total RNA was isolated from neuroblastoma or rhabdomyosarcoma cells that have been transiently transfected with different siRNAs or siCtrl for 48 h or 72 h and subjected to RNA-seq analysis as previously described [60]. Total RNA was extracted using the RNeasy Plus Mini Kit (Qiagen) according to the manufacturer’s instructions. TruSeq Stranded Total RNA LT Library Prep Kit or TruSeq Stranded mRNA Library Prep kit (Illumina, San Diego, California, United States of America) was used for preparing Strand-specific whole transcriptome sequencing libraries by following the manufacturer’s procedure. The Fastq files with paired-end reads were processed using Partek Flow. The raw reads are aligned using STAR (RRID:SCR_004463) and the aligned reads are quantified to the annotation model through Partek E/M. The normalization method used here is counts per million (CPM) through Partek Flow. The statistic analysis of normalized counts used GSA or ANOVA. To get T-scores, the normalized counts acquired from Partek Flow are exported and further analyzed using Parteck Genomics Suite v7.17. Statistical results of differentially expressed genes from Partek Flow were analyzed using QIAGEN’s IPA (QIAGEN) and GSEA. By default, the false discovery rate (FDR) less than 0.25 is significant in GSEA.

### ChIP-seq

ChIP-seq was performed using the ChIP-IT High Sensitivity kit (Active Motif, cat. 53040) as described previously [60]. Briefly, formaldehyde (1%, 13 min) fixed cells were sheared to achieve chromatin fragmented to a range of 200 to 700 bp using an Active Motif EpiShear Probe Sonicator. IMR32 cells that have been transiently transfected with different siRNAs that target different genes or negative control siRNA (siCtrl) for 72 h were used for ChIP-seq. IMR32 cells were sonicated at 25% amplitude, pulse for 20 s on and 30 s off for a total sonication “on” time of 16 min. Sheared chromatin samples were immunoprecipitated overnight at 4°C with antibodies targeting MYCN, G9a, WDR5, H3K27ac, H3K4me1, H3K4me3, and H3K27me3 (S12 Table). To compare the colocalization between MYCN and other proteins on the genome, ChIP-seq data from negative control siRNA transfected IMR32 cells were used. ChIP-seq DNA libraries were prepared by Frederick National Laboratory for Cancer Research sequencing facility. Libraries were multiplexed and sequenced using TruSeq ChIP Samples Prep Kit (75 cycles), cat. # IP-2-2-1012/1024 on an Illumina NextSeq machine, and 25,000,000 to 30,000,000 unique reads were generated per sample.

### ChIP-seq data processing

Previously published ChIP-seq datasets are downloaded for this study, which includes ChIP-seq datasets of MYCN that were generated in BE(2)C cells (GSE94822). As described previously [60], for the home generated ChIP-seq data, ChIP enriched DNA reads were mapped to reference the human genome (version hg19) using BWA (RRID:SCR_010910). Duplicate reads were infrequent but discarded.

ChIP-seq read density values were normalized per million mapped reads. High-confidence ChIP-seq peaks were called by MACS2 (https://github.com/taoliu/MACS) with the broad peak calling for H3K27me3, narrow peak for the rest proteins. Peaks from ChIP-seq of H3K27ac, H3K4me1, H3K4me3, H3K27me3, MYCN, WDR5, and G9a were selected based on *p*-value (*p* < 10^−5^ for G9a, *p* < 10^−7^ for the rest proteins). HOMER (RRID:SCR_010881) was used to annotate the distribution of peaks (such as enhancer, promoter, intronic, intergenic, exonic, etc.) and identify the known and de novo motifs.

The peak sets for MYCN, histone marks, and other proteins were further analyzed using the deepTools2 suite (v3.3.0) [61]. By using bamCoverage, peaks were normalized to reads per kilobase per million reads normalized read numbers (RPKM). Heatmaps and metagene plots of signal intensity of ChIP samples were generated using deepTools. Briefly, computeMatrix was used to calculate signal intensity scores per ChIP sample in a given genome region that was specified by a bed file. The output of computeMatrix was a matrix file of scores of 2 ChIP samples which was then used to generate the heatmaps using the plotHeatmap function and generate composite plot using the plotProfile function. For *k*-means clustering, the resulting matrix was *k*-means clustered and then visualized using plotHeatmap (—kmeans 2). For IGV sample track visualization, RPKM normalized coverage density maps (tdf files) were generated by extending reads to the average size and counting the number of reads mapped to each 25 bp window using igvtools [62].

ComputeMatrix function of the deepTools was used to generate a matrix of signal intensity of TFs of their peak centers (±500 bp, total 1,000 bp), as intensity scores in 10 bp bins. The matrix of signal intensity was further used to calculate the accumulated signal around each peak center, which was then used as the signal intensity for each TF binding site.

To find the unique and overlapped peaks between the binding sites of MYCN and its cofactors, R package, ChIPpeakAnno was applied. The function of “findOverlapsOfPeaks” was used with the “connectedPeaks” set to “min” [63].

The super-enhancers were identified using the ROSE2 (Rank Order of Super-Enhancers) software (https://github.com/BradnerLab/pipeline) using distal (>2,500 bp from TSS) H3K27ac peaks [64,65]. Enhancer constituents were stitched together if clustered within a distance of 12.5 kb. The enhancers were classified into typical and super-enhancers based on a cutoff at the inflection point in the rank ordered set (where tangent slope = 1) of the ChIP-seq signal (input normalized).

### ChIP-re-ChIP

ChIP-re-ChIP was performed by combining the ChIP-IT High Sensitivity kit (Active Motif, cat. 53040) and Re-ChIP-IT kit (Active Motif, cat. 53016). IMR32 cells were fixed and sheared to achieve chromatin fragmented to a range of 200 to 700 bp by following the manual of the ChIP-IT High Sensitivity kit. Next, ChIP-re-ChIP was performed by following the manual of the Re-ChIP-IT kit. The first ChIP was performed by using either anti-MYCN antibody or IgG control. The eluted chromatin acquired from the first MYCN ChIP reaction was used for the second ChIP by using anti-WDR5 antibody or IgG. ChIP-PCR was performed by using primers that recognize MYCN/WDR binding site within the *RPL38* gene promoter region (GRCh38.p14, Chr18:74203681–74203844). The sequences of the primer sets are: RPL38_F, TTTCGTCCTTTTCCCCGGTT; RPL38_F, AAATATCGGCCCCATCGCAC.

### Statistics

The statistical analyses used throughout this paper are specified in the appropriate results paragraphs and Methods sections. Additional statistical analyses were performed using standard two-tailed Student’s *t* test, one-way ANOVA, and the software GraphPad Prism (RRID:SCR_002798).

## Supporting information

S1 Fig MYCN maintains malignant NB cell identity through directly activating canonical MYC target genes and suppressing neuronal differentiation genes (supplementary to Fig 1). (**A**) The knockdown of MYCN in IMR32 cells using 2 different siRNAs for 7 days results in an increase of neurite length and axon formation shown by the phase-contrast images. (B–D) The knockdown of *MYCN* in BE(2)C cells results in a decrease in cell number and neurite length. (E–G) The knockdown of *MYCN* in KCNR cells results in a decrease in cell number and neurite length. (H–J) The knockdown of *MYCN* in LAN5 cells results in a decrease of cell number and neurite length. (K) The expression of MYCN protein in SHEP cells that stably transfected with doxycycline (Dox) inducible *MYCN* expression construct (SHEPtetMYCN) is detected by western blot after Dox treatment. (L) The induction of *MYCN* in SHEP cells results in a change in cell morphology. (M) and (N) The overexpression of *MYCN* in SHEP cells results in an increase of colony formation in soft agar shown by both the crystal violet staining and colony count. (O) Gene set enrichment analysis (GSEA) of the RNA-seq data shows that the knockdown of *MYCN* in IMR32 cells for 72 h results in a negative enrichment of hallmark MYC targets and canonical MYC target genes that are involved in ribosome biogenesis, as well as a positive enrichment of neuron markers and genes regulate synaptic transmission. (P) GSEA of the RNA-seq data shows that the knockdown of *MYCN* using a different *MYCN* siRNA (*siMYCN_4*) in IMR32 cells for 72 h results in a negative enrichment of MYC target genes and ribosome biogenesis genes, and a positive enrichment of neuron markers and synaptic transmission genes. (Q) GSEA of the RNA-seq data shows that the knockdown of *MYCN* in KCNR cells for 72 h results in a negative enrichment of MYC target genes and ribosome biogenesis genes, and a positive enrichment of neuron markers and synaptic transmission genes. (R) GSEA of the RNA-seq data shows that the knockdown of *MYCN* in LAN5 cells for 72 h results in a negative enrichment of MYC target genes and ribosome biogenesis genes, and a positive enrichment of neuron markers and synaptic transmission genes. (S) GSEA of the RNA-seq data shows that the overexpression of *MYCN* in SHEP cells results in a positive enrichment hallmark of MYC targets and canonical MYC targets that are involved in ribosome biogenesis, as well as a negative enrichment of synaptic transmission genes and synapse assembly genes. (T) GREAT peak distribution analysis shows that the majority of MYCN peaks belonging to cluster 1 of Fig 1E are within 5 kb from the TSS (left panel), while the majority of MYCN peaks belonging to cluster 2 of Fig 1E are over 5 kb from the TSS (right panel). (U) GREAT GO analysis of MYCN binding sites associated genes in BE(2)C cells show that MYCN-bound promoter associated genes are enriched in canonical MYC target genes such as genes that regulate RNA processing and ribosome biogenesis, while MYCN-bound distal regulatory regions associated genes were enriched in nervous system development. (V) GREAT GO analysis indicates that MYCN-bound promoters associated genes up-regulated after the knockdown of MYCN are enriched in pons development and axon regeneration. (W) GREAT GO analysis indicates that MYCN-bound enhancers associated genes down-regulated after the knockdown of MYCN are enriched in chordate embryonic development. (X) Signal tracks show that MYCN colocalizes with H3K27ac and H3K4me3 on the promoters but not the enhancers of cell cycle genes *CDK4* and *CCNA2*, as well as metabolic genes *ODC1*, *LDHA*, *PKM*, *GLS*, and *PRIM1*. (Y) GSEA of the RNA-seq data shows that the knockdown of *MYCN* in IMR32 cells for 72 h results in a negative enrichment of cell cycle progression genes. (Y) GSEA of the RNA-seq data shows that the knockdown of *MYCN* in IMR32 cells for 72 h results in a negative enrichment of genes involved in metabolic processes. The data underlying the graphs in the figure are shown in S1 data.
(PDF)

S2 Fig MYCN binds to the promoters to activate canonical MYC targets and binds to the enhancers to repress muscle differentiation genes in RMS (supplementary to Fig 2). (A) The protein levels of MYCN in IMR32, RH4, and 293T cells detected by western blot assay. (**B**) The knockdown of *MYCN* in RH4 cells results in a decrease of MYCN at the protein levels detected by western blot assay. (**C**) The knockdown of *MYCN* in RH4 cells results in a decrease of cell number based on IncuCyte cell confluence assay and (**D**) the cell imaging. (**E**) GSEA of the RNA-seq data shows that the knockdown of *MYCN* in RH cells for 72 h results in a negative enrichment of cell cycle progression genes and MYC targets. The data underlying the graphs in the figure are shown in S1 Data.
(PDF)

S3 Fig MYCN regulates regional but not global histone modification (supplementary to Fig 3). (**A**) Heatmap of MYCN and histone marks ChIP-seq around MYCN binding sites (±3 kb) before and after knocking down MYCN in IMR32 cells. (**B**) Heatmap of MYCN and histone marks ChIP-seq around TSS (±3 kb) of the whole genome before and after knocking down MYCN in IMR32 cells. (**C**) *k*-Means clustering of MYCN and histone marks ChIP-seq around MYCN binding sites in *MYCN* knockdown IMR32 shows that MYCN binds to proximal regulatory elements containing active promoters that are marked by H3K27ac and H3K4me3 signals, and distal regulatory elements containing enhancers that are marked by H3K4me1 and H3K27ac signals. (**D**) In *MYCN* knockdown IMR32 cells, the percentage of MYCN peaks within enhancers is significantly decreased, while the percentage of MYCN peaks within promoters is significantly increased compared to the MYCN peak distribution in *siCtrl*-transfected cells based on chi-square test (*p* < 0.05).
(PDF)

S4 Fig Genome-wide colocalization of MYCN and its cofactors (supplementary to Fig 4). (**A**) The immunoprecipitation of MYCN using 2 different MYCN antibodies is detected by western blot analysis. (**B**) Annotation of the subcellular localization of MYCN interactors by using ingenuity pathway analysis tool. (**C**) DAVID functional annotation of MYCN nuclear protein partners. (**D**) The pulldown of WDR5 and G9a after co-IP of MYCN is detected by western blot analysis.
(PDF)

S5 Fig Silencing of *MYCN* selectively alters genomic DNA binding of its cofactors (supplementary to Fig 5). (**A**) Signal tracks show that the knockdown of *MYCN* results in a decrease of MYCN and WDR5 signals at the promoter of *RPL8* gene and *PVR* gene. (**B**) Signal tracks show that the knockdown of *MYCN* results in a decrease of MYCN and G9a signals within the intron of the *KCNK3* gene. **(C)** IPA of the G9a ChIP-seq data shows that the genes associated with G9a binding sites with stable ChIP-seq signal (within 1.1-fold change after the silencing of *MYCN*) are enriched in nervous system development. (**D**) IPA of the G9a ChIP-seq data shows that the genes associated with G9a binding sites increased ChIP-seq signal (>1.2-fold increase after the silencing of *MYCN*) are enriched in organismal development. The data underlying the graphs in the figure are shown in S1 Data.
(PDF)

S6 Fig MYCN cofactors assist MYCN to bind to DNA (supplementary to Fig 6). (**A**) Metagene plots show that the knockdown of *WDR5* results in a decrease of average WDR5 and MYCN signal at the WDR5 peak center when focused on MYCN-bound promoters defined in Fig 1E. (**B**) Metagene plots show that the knockdown of *WDR5* results in a decrease of average WDR5 and MYCN signal at the WDR5 peak center when focused on MYCN-bound enhancers defined in Fig 1E. (**C**) Metagene plots show that the genomic loci with subtle decrease (<10% at the summit) in average WDR5 ChIP-seq signal intensity at the WDR5 peak center only showed subtle decrease (<10% at the summit) in MYCN ChIP-seq signal intensity. (**D**) Signal tracks show that the knockdown of *WDR5* does not result in a decrease of WDR5 and MYCN signals at the promoter of *NAT9* gene and *TMEM* gene. (**E**) ChIP-PCR results reveal that both the first ChIP with the anti-MYCN antibody (lane 3) and the second re-ChIP with the anti-WDR5 antibody (lane 4) but not the IgG control (lane 2 and 5) pulled down DNA fragments within the *RPL38* promoter region. The first ChIP was performed by using either IgG control (lane 2) or an anti-MYCN antibody (lane 3). The eluted chromatin acquired from the first MYCN ChIP reaction was used for the second ChIP by using an anti-WDR5 antibody (lane 4) or IgG control (lane 5). The data underlying the graphs in the figure are shown in S1 Data.
(PDF)

S7 Fig The depletion of MYCN cofactors antagonizes MYCN-mediated gene transcription regulation (supplementary to Fig 7). (**A**) Western blot analysis shows that the knockdown of WDR5 and G9a using siRNAs for 48 h results in a decrease of their expression at protein levels. (**B**) GSEA shows that the silencing of *MYCN* for 48 h results in a significant negative enrichment of genes involved in ribosome biogenesis, RNA processing, ribosome formation, and protein synthesis. (**C**) GSEA shows that the silencing of *MYCN* results in a significant positive enrichment of genes involved in axon development, neuron differentiation, glutamatergic synapse, and neuron projection guidance. (**D**) GSEA shows that the silencing of *G9a* results in a significant positive enrichment of genes involved in glutamatergic synapse and neuron projection guidance that are activated by MYCN. (**E**) DepMap CRISPR library screen data analysis (https://depmap.org/portal/) shows that WDR5 or G9a is essential for a majority of the neuroblastoma cell lines to survive or proliferate based on the CRISPR dependence score. Note: *MYCN*_SC, *MYCN* single copy NB cell lines; *MYCN*_AMP, *MYCN* amplified NB cell lines. (**F**) Genetic silencing of *WDR5* or *G9a* using siRNAs in IMR32 cells resulted in a decrease in cell proliferation shown by the IncuCyte confluence assay. The data underlying the graphs in the figure are shown in S1 Data.
(PDF)

S1 Table Genes regulated by MYCN in NB cell lines IMR32, KCNR, LAN5, SHEP, and RMS cell line RH4. (XLSX)

S2 Table GREAT GO analysis of enhancers with or without MYCN binding, as well as MYCN-bound promoters in IMR32 cells. (XLSX)

S3 Table GREAT GO analysis of enhancers with or without MYCN binding, as well as MYCN-bound promoters in RH4 cells. (XLSX)

S4 Table MYCN protein partners identified in NB cells. (XLSX)

S5 Table WDR5 ChIP-seq peaks identified in *siCtrl* and *siMYCN* transfected NB cells. (XLSX)

S6 Table Genes commonly and differentially regulated by MYCN and WDR5 in IMR32 cells. (XLSX)

S7 Table Ingenuity Pathway Analysis of genes commonly and differentially regulated by MYCN and WDR5 in IMR32 cells. (XLSX)

S8 Table G9a ChIP-seq peaks identified in *siCtrl* and *siMYCN* transfected NB cells. (XLSX)

S9 Table WDR5 ChIP-seq peaks identified in *siCtrl* and *siWDR5* transfected NB cells. (XLSX)

S10 Table GREAT GO analysis of WDR5 and MYCN overlapped ChIP-seq peaks with >2.5-fold decrease of WDR5 signal intensity after the silencing of *WDR5*. (XLSX)

S11 Table Gene sets regulated by MYCN derived from GSEA of MYCN regulated transcriptome. (XLSX)

S12 Table List of antibodies used in western blot, co-IP, and ChIP-seq. (XLSX)

S1 Data Excel spreadsheet containing, in separate sheets, the underlying numerical data for figure panels 1B, 1C, 1H, 1L, 1M, 2D, 2H, 2I, 3A, 3B, 3C, 3D, 3E, 3F, 4G, 5B, 5C, 5D, 5E, 6B, 6C, 6E, 7C, 7D, 7E, 7F, 7G, 7H, as well as supplementary figure panels S1B, S1C, S1E, S1F, S1H, S1I, S1N, S1T, S2C, S5C, S5D, S6A, S6B, S6C, S7E, and S7F. (XLSX)

S1 Raw Images This file contains all raw images of blots and gels. (PDF)

## Abbreviations

co-IP: co-immunoprecipitation
CPM: counts per million
FDR: false discovery rate
GO: Gene Ontology
GREAT: genomic regions enrichment of annotation tool
GSEA: gene set enrichment analysis
IPA: Ingenuity Pathway Analysis
NB: neuroblastoma
RMS: rhabdomyosarcoma
TF: transcription factor
TSS: transcription start site
