# Supplementary material for: MYCN drives oncogenesis by cooperating with the histone methyltransferase G9a and the WDR5 adaptor to orchestrate global gene transcription
Source: PLoS Biol. 2024 Mar 28;22(3):e3002240. doi: 10.1371/journal.pbio.3002240 (PMC11003700; doi:10.1371/journal.pbio.3002240)

Supplementary Fig. 1

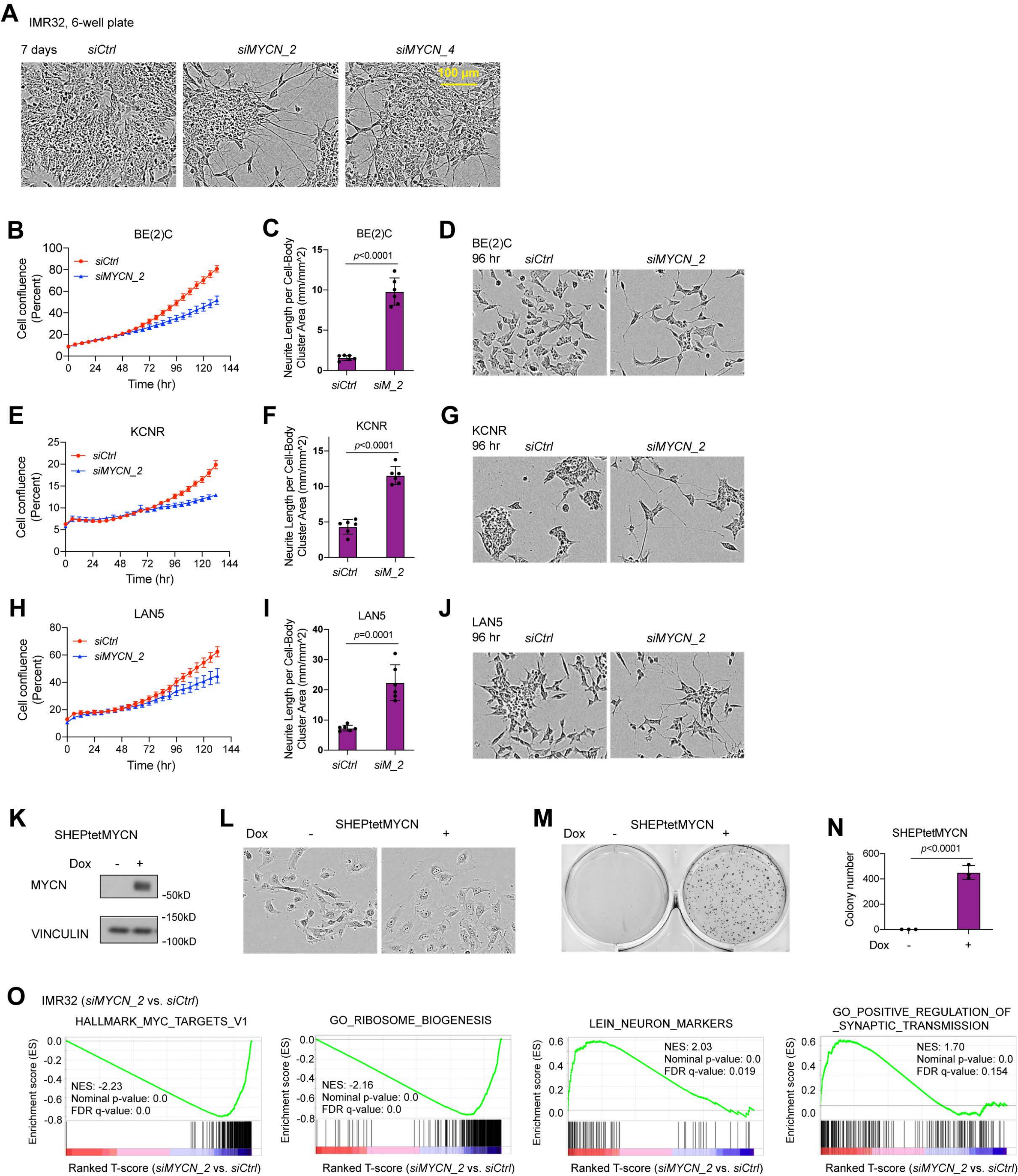

Supplementary Fig. 1 continued

P IMR32 (*siMYCN_4* vs. *siCtrl*)

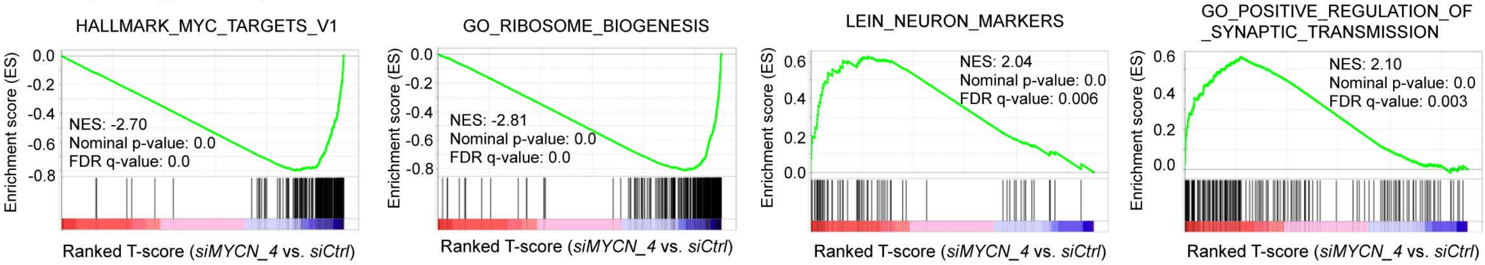

Q KCNR (*siMYCN_2* vs. *siCtrl*)

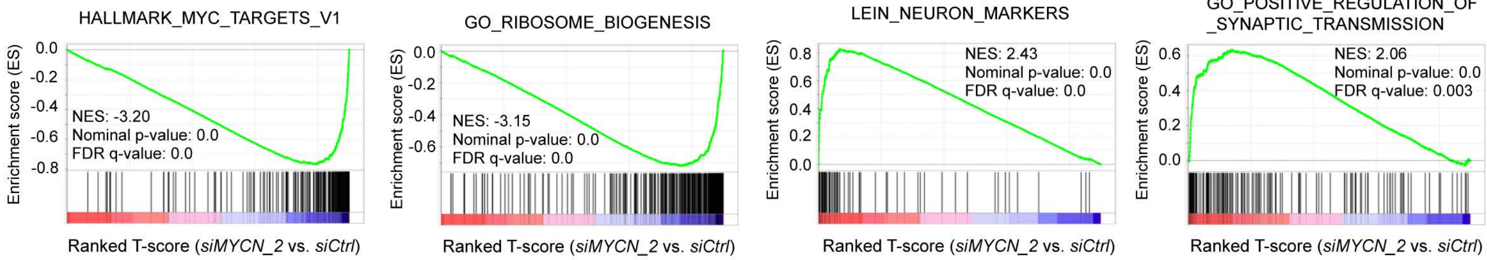

R LAN5 (*siMYCN_2* vs. *siCtrl*)

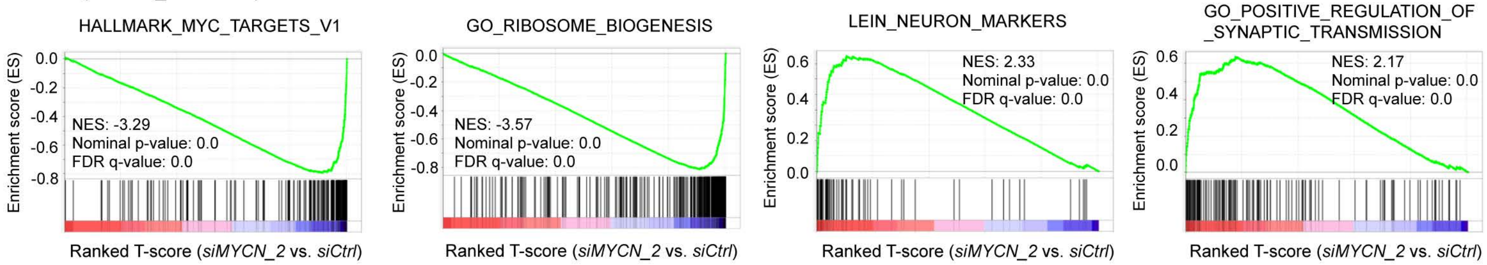

S SHEPtetMYCN

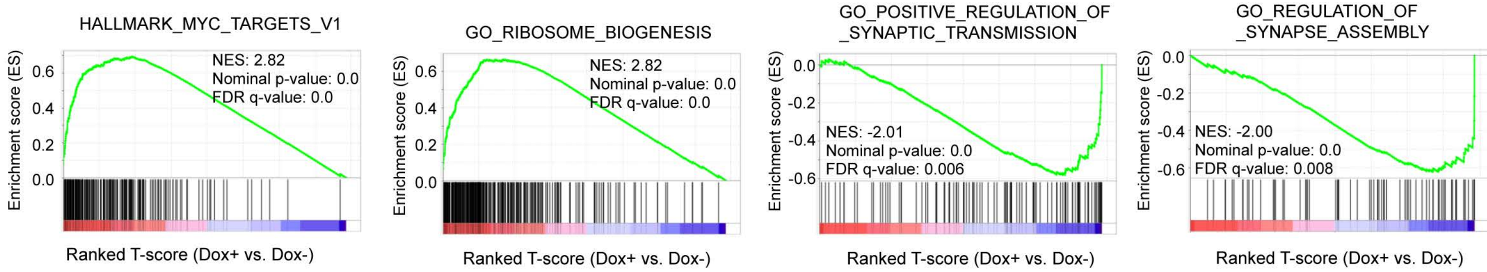

T

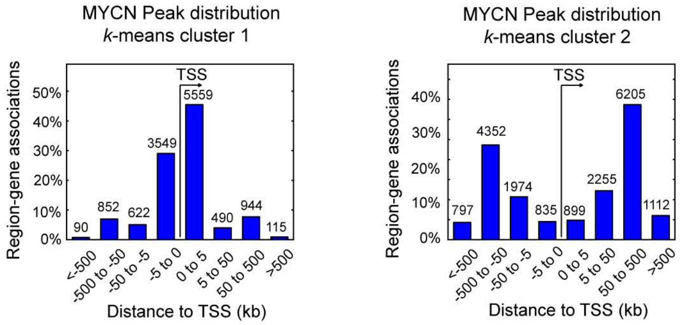

U BE(2)C

Promoters

GREAT GO biological process	Binom FDR Q-Val
ncRNA metabolic process	7.67E-54
ribonucleoprotein complex biogenesis	7.15E-54
ribosome biogenesis	1.56E-51
ncRNA processing	6.64E-49
RNA processing	3.55E-47

Distal regulatory regions

GREAT GO biological process	Binom FDR Q-Val
Cartilage condensation	9.11E-22
Autonomic nervous system development	2.31E-16
Embryonic digit morphogenesis	4.55E-16
Sympathetic nervous system development	1.01E-15
Noradrenergic neuron differentiation	5.51E-15

Supplementary Fig. 1 continued

V IMR32

MYCN-bound promoters associated genes up-regulated after MYCN knockdown (<-1.5-fold, q<0.05)

GREAT GO biological process	Binom FDR Q-Val
Pons development	5.65E-6
Response to axon injury	7.76E-4
Axon regeration	1.25E-3
Endoplasmic reticulum tubular network organization	2.97E-3
Regulation of cardiac muscle contraction by calcium ion signaling	2.69E-3

W IMR32

MYCN-bound enhancers associated genes down-regulated after MYCN knockdown (>1.5-fold, q<0.05)

GREAT GO biological process	Binom FDR Q-Val
Chordate embryonic development	3.15E-6
Embryo development ending in birth or egg hatching	3.56E-6

X

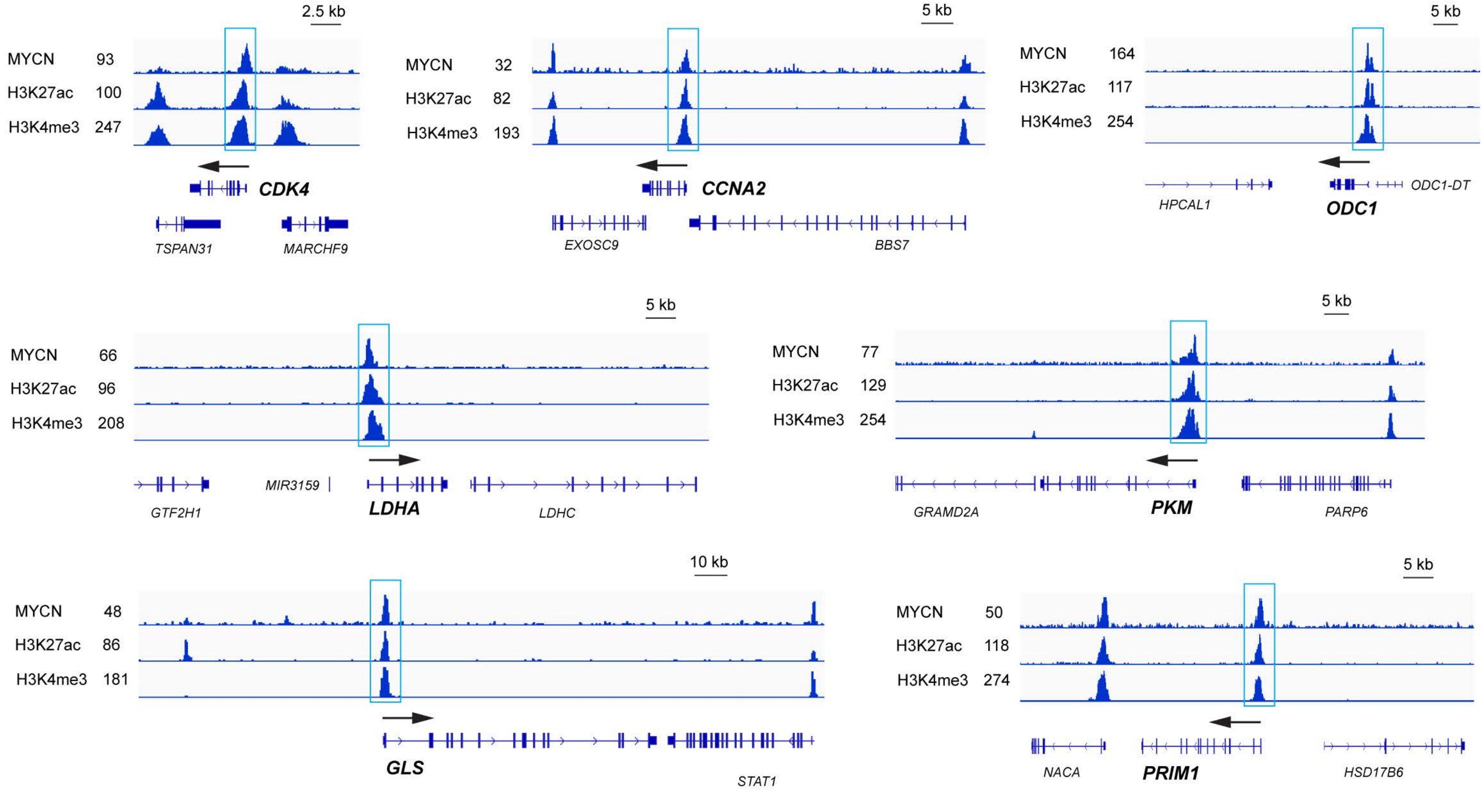

Y IMR32 siMYCN-2_72h

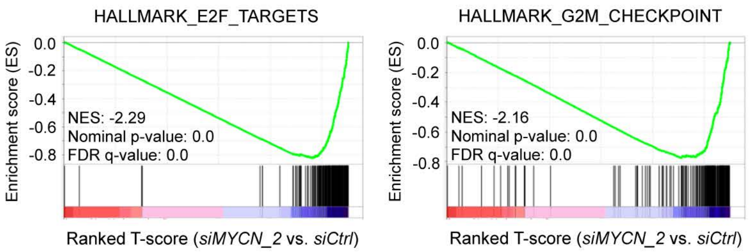

Z IMR32 siMYCN-2_72h

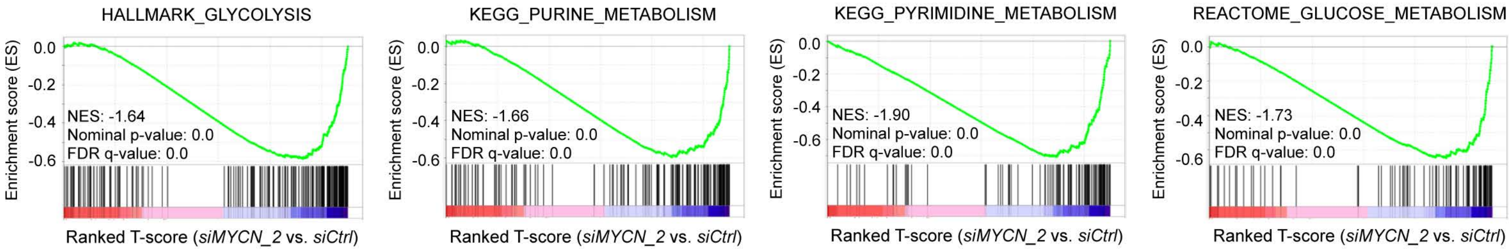

Supplement: S1 Fig — (A) The knockdown of MYCN in IMR32 cells using 2 different siRNAs for 7 days results in an increase of neurite length and axon formation shown by the phase-contrast images. (B–D) The knockdown of MYCN in BE(2)C cells results in a decrease in cell number and neurite length. (E–G) The knockdown of MYCN in KCNR cells results in a decrease in cell number and neurite length. (H–J) The knockdown of MYCN in LAN5 cells results in a decrease of cell number and neurite length. (K) The expression of MYCN protein in SHEP cells that stably transfected with doxycycline (Dox) inducible MYCN expression construct (SHEPtetMYCN) is detected by western blot after Dox treatment. (L) The induction of MYCN in SHEP cells results in a change in cell morphology. (M) and (N) The overexpression of MYCN in SHEP cells results in an increase of colony formation in soft agar shown by both the crystal violet staining and colony count. (O) Gene set enrichment analysis (GSEA) of the RNA-seq data shows that the knockdown of MYCN in IMR32 cells for 72 h results in a negative enrichment of hallmark MYC targets and canonical MYC target genes that are involved in ribosome biogenesis, as well as a positive enrichment of neuron markers and genes regulate synaptic transmission. (P) GSEA of the RNA-seq data shows that the knockdown of MYCN using a different MYCN siRNA (siMYCN_4) in IMR32 cells for 72 h results in a negative enrichment of MYC target genes and ribosome biogenesis genes, and a positive enrichment of neuron markers and synaptic transmission genes. (Q) GSEA of the RNA-seq data shows that the knockdown of MYCN in KCNR cells for 72 h results in a negative enrichment of MYC target genes and ribosome biogenesis genes, and a positive enrichment of neuron markers and synaptic transmission genes. (R) GSEA of the RNA-seq data shows that the knockdown of MYCN in LAN5 cells for 72 h results in a negative enrichment of MYC target genes and ribosome biogenesis genes, and a positive enrichment of neuron marker [file pbio.3002240.s001.pdf]
