# Supplementary figures and images for: MYCN drives oncogenesis by cooperating with the histone methyltransferase G9a and the WDR5 adaptor to orchestrate global gene transcription
Source: PLoS Biol. 2024 Mar 28;22(3):e3002240. doi: 10.1371/journal.pbio.3002240 (PMC11003700; doi:10.1371/journal.pbio.3002240)

# Supplementary Fig. 2

**A**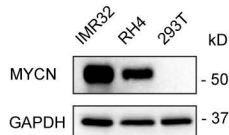**B**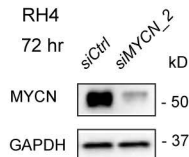**C**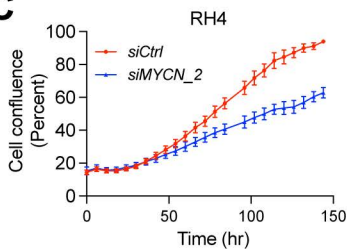**D**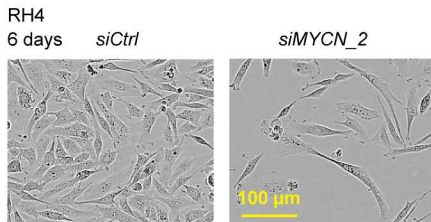**E**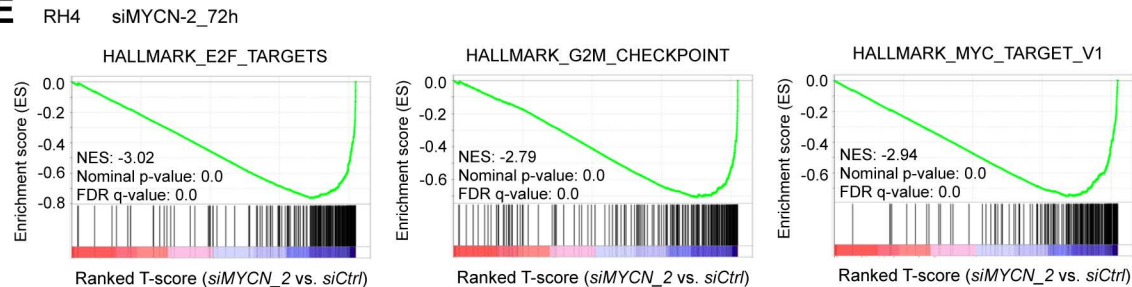

Supplement: S2 Fig — (A) The protein levels of MYCN in IMR32, RH4, and 293T cells detected by western blot assay. (B) The knockdown of MYCN in RH4 cells results in a decrease of MYCN at the protein levels detected by western blot assay. (C) The knockdown of MYCN in RH4 cells results in a decrease of cell number based on IncuCyte cell confluence assay and (D) the cell imaging. (E) GSEA of the RNA-seq data shows that the knockdown of MYCN in RH cells for 72 h results in a negative enrichment of cell cycle progression genes and MYC targets. The data underlying the graphs in the figure are shown in S1 Data. (PDF) [file pbio.3002240.s002.pdf]

# Supplementary Fig. 3

**A**

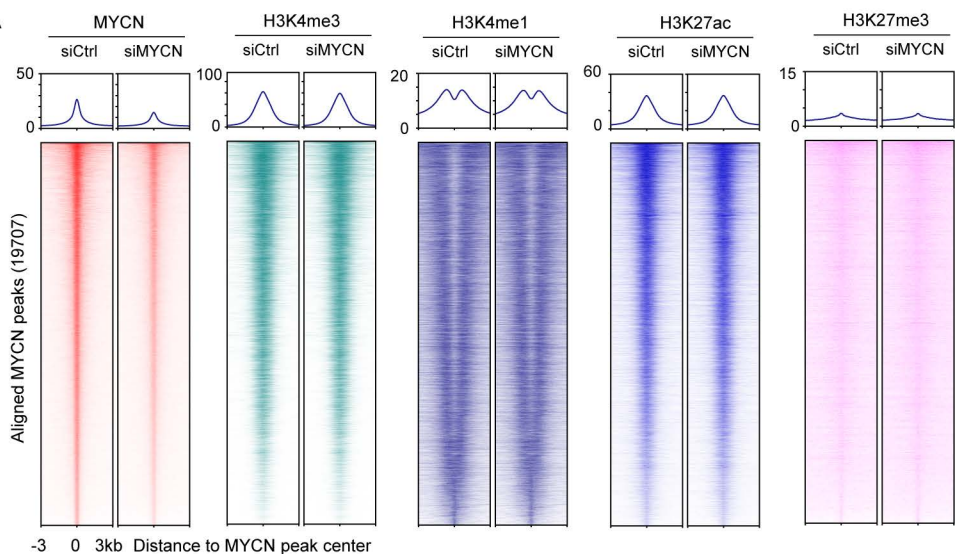

**B**

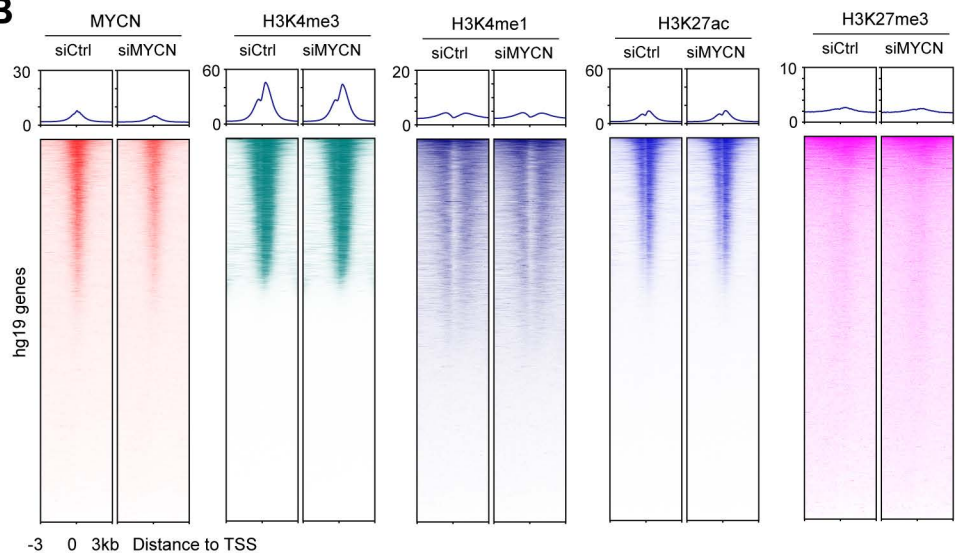

**C**

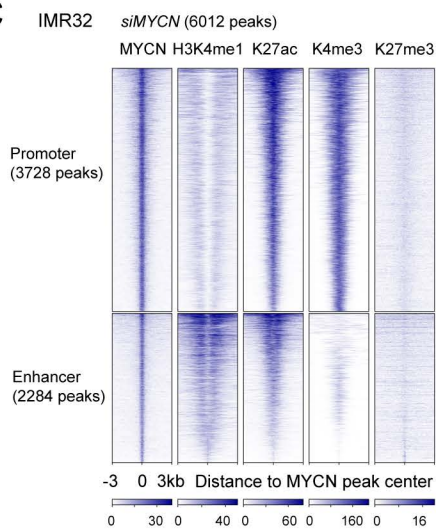

**D**

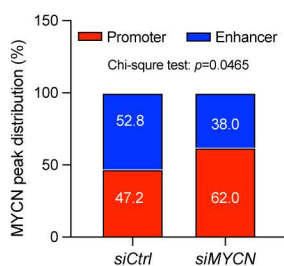

Supplement: S3 Fig — (A) Heatmap of MYCN and histone marks ChIP-seq around MYCN binding sites (±3 kb) before and after knocking down MYCN in IMR32 cells. (B) Heatmap of MYCN and histone marks ChIP-seq around TSS (±3 kb) of the whole genome before and after knocking down MYCN in IMR32 cells. (C) k-Means clustering of MYCN and histone marks ChIP-seq around MYCN binding sites in MYCN knockdown IMR32 shows that MYCN binds to proximal regulatory elements containing active promoters that are marked by H3K27ac and H3K4me3 signals, and distal regulatory elements containing enhancers that are marked by H3K4me1 and H3K27ac signals. (D) In MYCN knockdown IMR32 cells, the percentage of MYCN peaks within enhancers is significantly decreased, while the percentage of MYCN peaks within promoters is significantly increased compared to the MYCN peak distribution in siCtrl-transfected cells based on chi-square test (p < 0.05). (PDF) [file pbio.3002240.s003.pdf]

# Supplementary Fig. 7

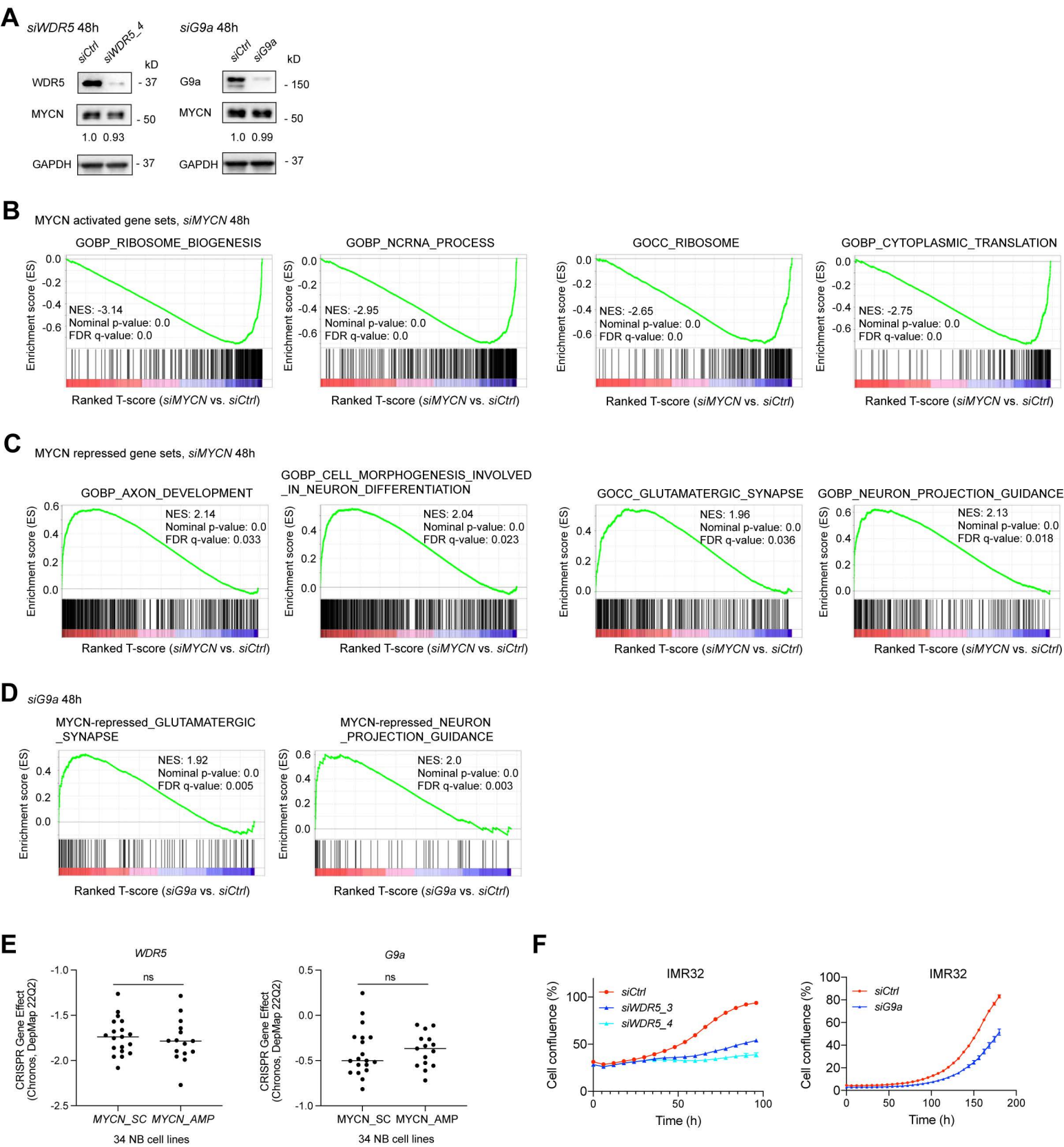

Supplement: S7 Fig — (A) Western blot analysis shows that the knockdown of WDR5 and G9a using siRNAs for 48 h results in a decrease of their expression at protein levels. (B) GSEA shows that the silencing of MYCN for 48 h results in a significant negative enrichment of genes involved in ribosome biogenesis, RNA processing, ribosome formation, and protein synthesis. (C) GSEA shows that the silencing of MYCN results in a significant positive enrichment of genes involved in axon development, neuron differentiation, glutamatergic synapse, and neuron projection guidance. (D) GSEA shows that the silencing of G9a results in a significant positive enrichment of genes involved in glutamatergic synapse and neuron projection guidance that are activated by MYCN. (E) DepMap CRISPR library screen data analysis (https://depmap.org/portal/) shows that WDR5 or G9a is essential for a majority of the neuroblastoma cell lines to survive or proliferate based on the CRISPR dependence score. Note: MYCN_SC, MYCN single copy NB cell lines; MYCN_AMP, MYCN amplified NB cell lines. (F) Genetic silencing of WDR5 or G9a using siRNAs in IMR32 cells resulted in a decrease in cell proliferation shown by the IncuCyte confluence assay. The data underlying the graphs in the figure are shown in S1 Data. (PDF) [file pbio.3002240.s007.pdf]
