# Supplementary material for: MYCN drives oncogenesis by cooperating with the histone methyltransferase G9a and the WDR5 adaptor to orchestrate global gene transcription
Source: PLoS Biol. 2024 Mar 28;22(3):e3002240. doi: 10.1371/journal.pbio.3002240 (PMC11003700; doi:10.1371/journal.pbio.3002240)

Supplementary Fig. 4

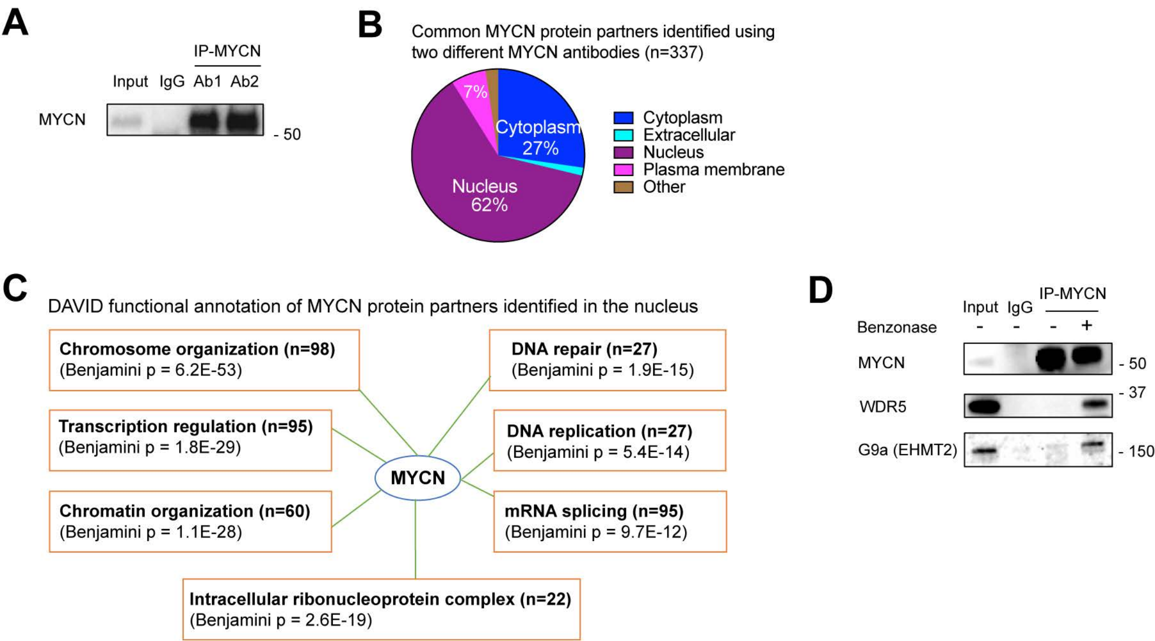

Supplement: S4 Fig — (A) The immunoprecipitation of MYCN using 2 different MYCN antibodies is detected by western blot analysis. (B) Annotation of the subcellular localization of MYCN interactors by using ingenuity pathway analysis tool. (C) DAVID functional annotation of MYCN nuclear protein partners. (D) The pulldown of WDR5 and G9a after co-IP of MYCN is detected by western blot analysis. (PDF) [file pbio.3002240.s004.pdf]
