# Supplementary material for: MYCN drives oncogenesis by cooperating with the histone methyltransferase G9a and the WDR5 adaptor to orchestrate global gene transcription
Source: PLoS Biol. 2024 Mar 28;22(3):e3002240. doi: 10.1371/journal.pbio.3002240 (PMC11003700; doi:10.1371/journal.pbio.3002240)

# Supplementary Fig. 5

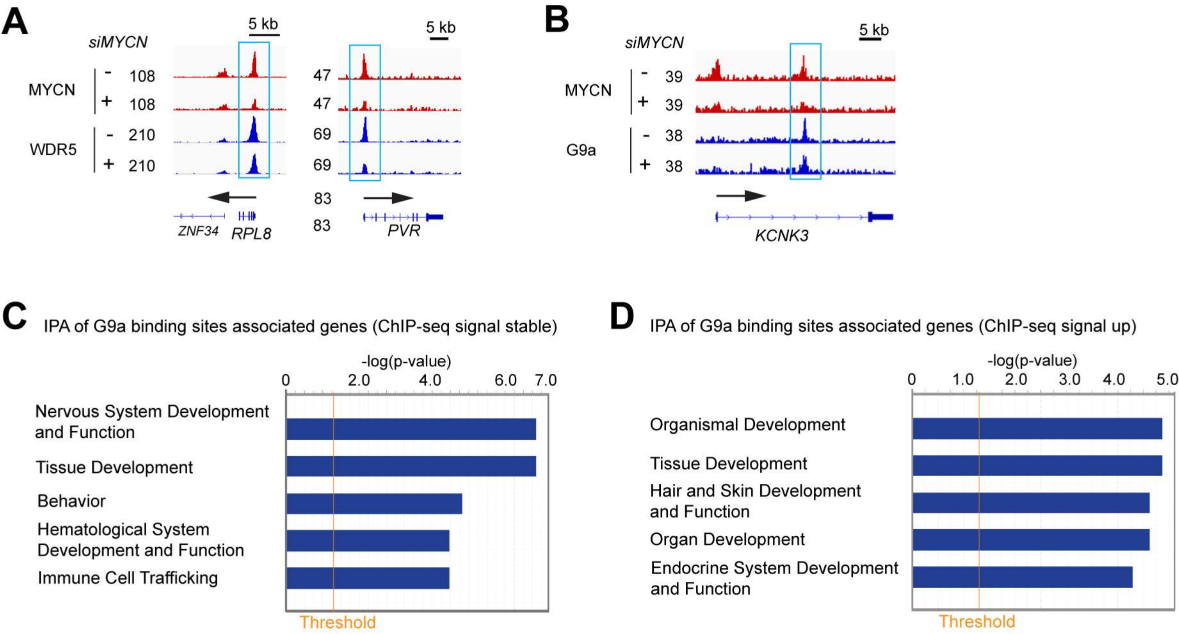

Supplement: S5 Fig — (A) Signal tracks show that the knockdown of MYCN results in a decrease of MYCN and WDR5 signals at the promoter of RPL8 gene and PVR gene. (B) Signal tracks show that the knockdown of MYCN results in a decrease of MYCN and G9a signals within the intron of the KCNK3 gene. (C) IPA of the G9a ChIP-seq data shows that the genes associated with G9a binding sites with stable ChIP-seq signal (within 1.1-fold change after the silencing of MYCN) are enriched in nervous system development. (D) IPA of the G9a ChIP-seq data shows that the genes associated with G9a binding sites increased ChIP-seq signal (>1.2-fold increase after the silencing of MYCN) are enriched in organismal development. The data underlying the graphs in the figure are shown in S1 Data. (PDF) [file pbio.3002240.s005.pdf]
