# Supplementary material for: MYCN drives oncogenesis by cooperating with the histone methyltransferase G9a and the WDR5 adaptor to orchestrate global gene transcription
Source: PLoS Biol. 2024 Mar 28;22(3):e3002240. doi: 10.1371/journal.pbio.3002240 (PMC11003700; doi:10.1371/journal.pbio.3002240)

Supplementary Fig. 6

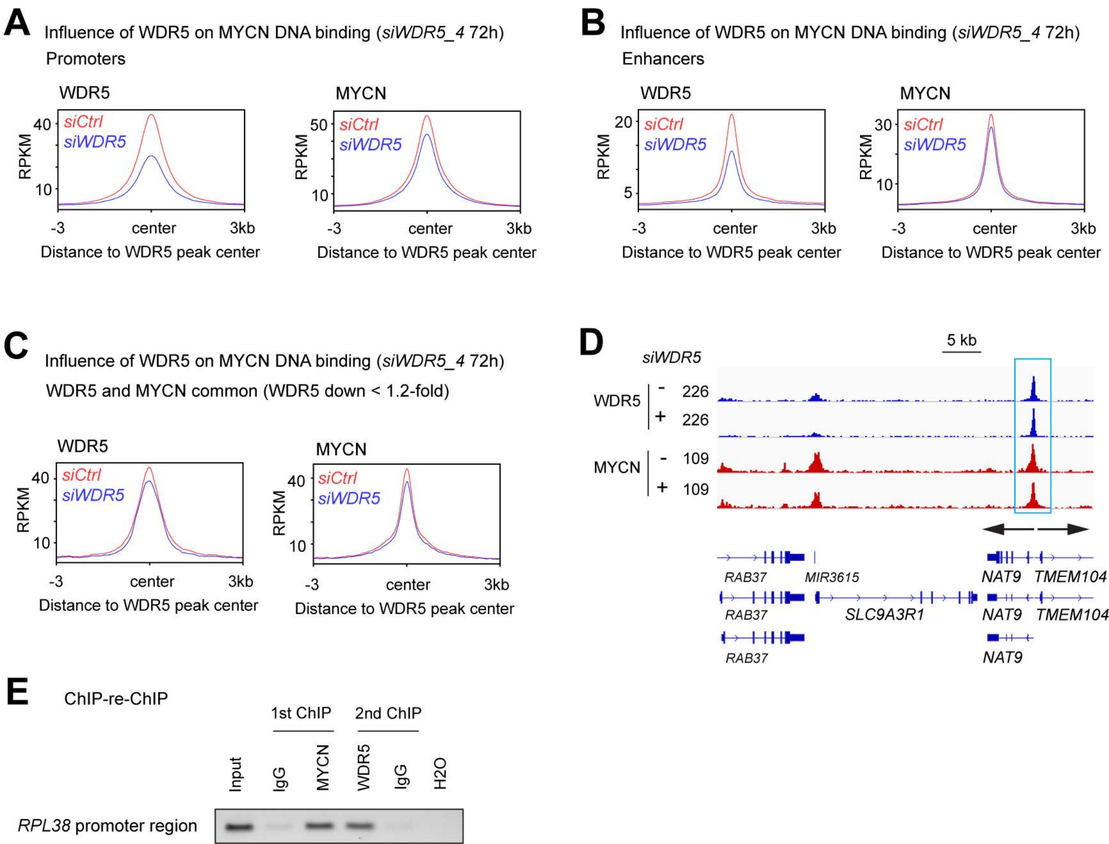

Supplement: S6 Fig — (A) Metagene plots show that the knockdown of WDR5 results in a decrease of average WDR5 and MYCN signal at the WDR5 peak center when focused on MYCN-bound promoters defined in Fig 1E. (B) Metagene plots show that the knockdown of WDR5 results in a decrease of average WDR5 and MYCN signal at the WDR5 peak center when focused on MYCN-bound enhancers defined in Fig 1E. (C) Metagene plots show that the genomic loci with subtle decrease (<10% at the summit) in average WDR5 ChIP-seq signal intensity at the WDR5 peak center only showed subtle decrease (<10% at the summit) in MYCN ChIP-seq signal intensity. (D) Signal tracks show that the knockdown of WDR5 does not result in a decrease of WDR5 and MYCN signals at the promoter of NAT9 gene and TMEM gene. (E) ChIP-PCR results reveal that both the first ChIP with the anti-MYCN antibody (lane 3) and the second re-ChIP with the anti-WDR5 antibody (lane 4) but not the IgG control (lane 2 and 5) pulled down DNA fragments within the RPL38 promoter region. The first ChIP was performed by using either IgG control (lane 2) or an anti-MYCN antibody (lane 3). The eluted chromatin acquired from the first MYCN ChIP reaction was used for the second ChIP by using an anti-WDR5 antibody (lane 4) or IgG control (lane 5). The data underlying the graphs in the figure are shown in S1 Data. (PDF) [file pbio.3002240.s006.pdf]
