# Supplementary material for: MYCN drives oncogenesis by cooperating with the histone methyltransferase G9a and the WDR5 adaptor to orchestrate global gene transcription
Source: PLoS Biol. 2024 Mar 28;22(3):e3002240. doi: 10.1371/journal.pbio.3002240 (PMC11003700; doi:10.1371/journal.pbio.3002240)

Raw immunoblots

Related to Fig. 1A

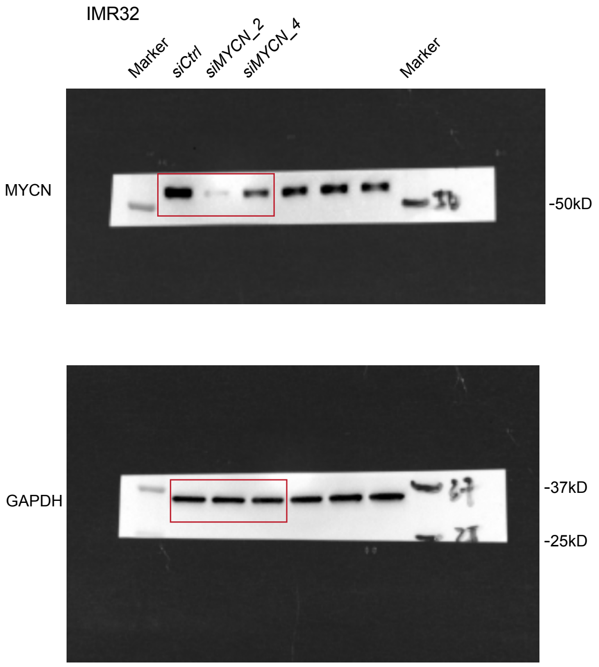

Raw gel

Related to Fig. 4A

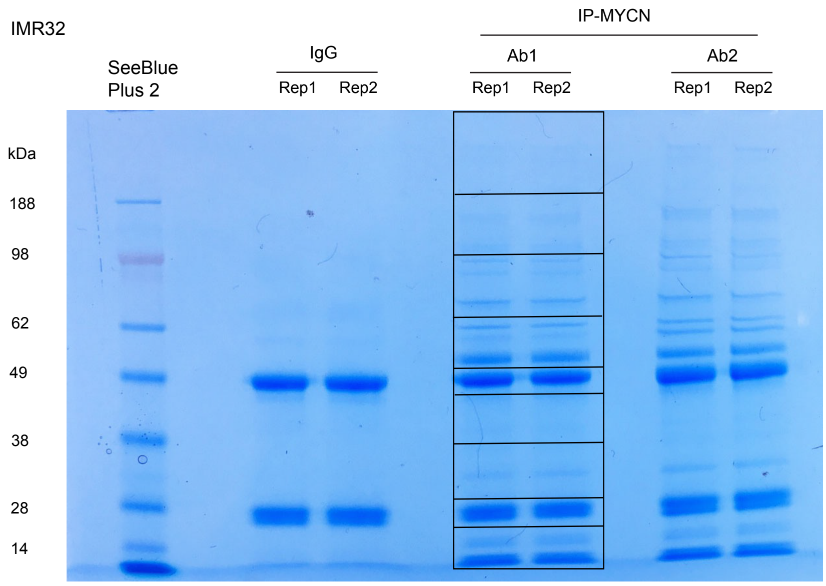

Black boxes indicate the manner in which gels were being cut for mass-spectrometry.
The three groups of samples were cut the same way.

Raw immunoblots

Related to Fig. 5A

Related to Fig. 5A, the left panel

IMR32

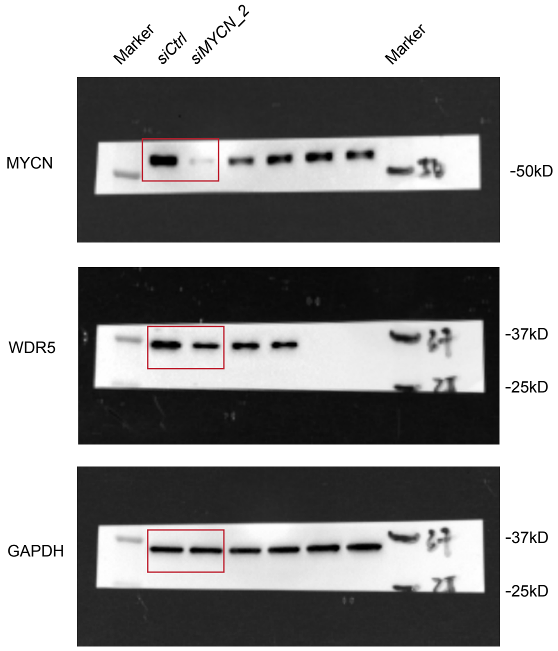

Related to Fig. 5A, the right panel

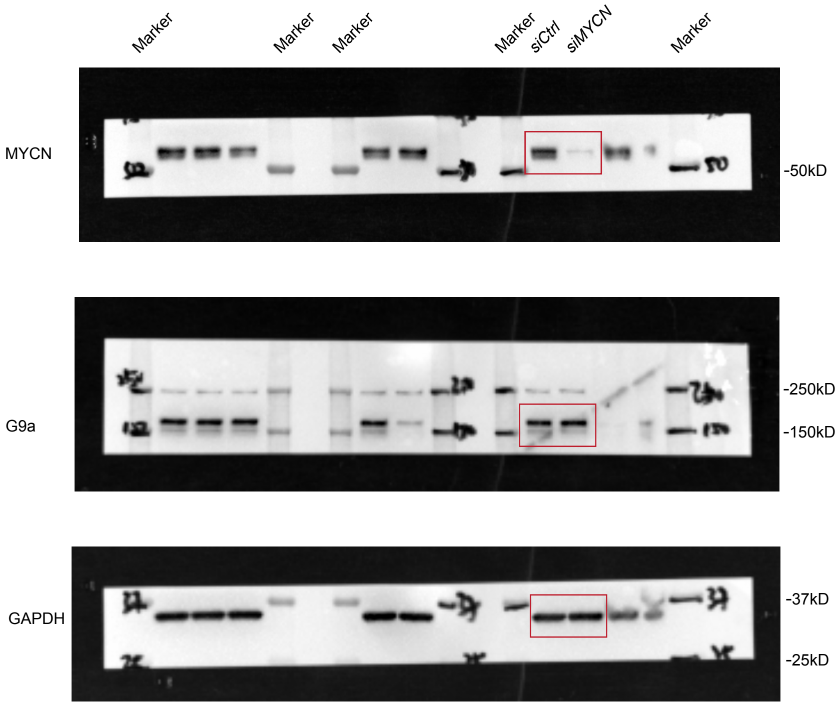

Raw immunoblots

Related to Fig. 6A

Marker siCtrl siWDR5_3 siWDR5_4 Marker

WDR5

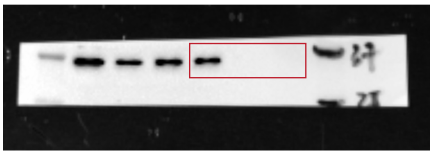

-37kD
-25kD

MYCN

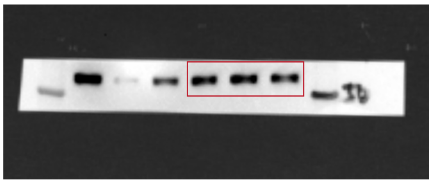

-50kD

GAPDH

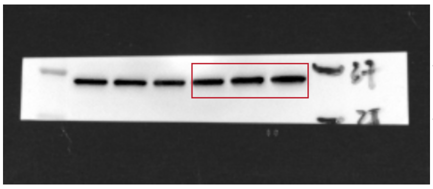

-37kD
-25kD

Raw immunoblots

Related to Fig. S1K

SHEPtetMYCN

Dox - + Marker

MYCN

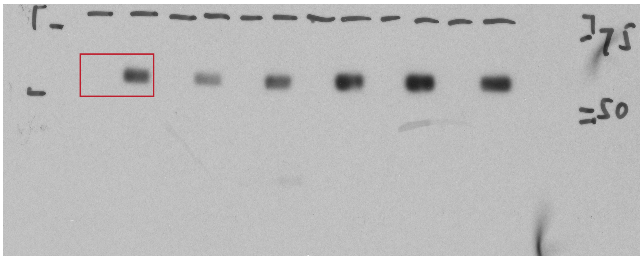

-75kD

-50kD

Dox - + Marker

VINCULIN

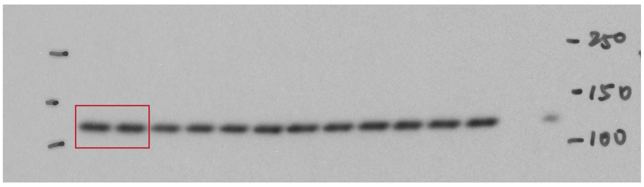

-250kD

-150kD

-100kD

Raw immunoblots

Related to Fig. S2A

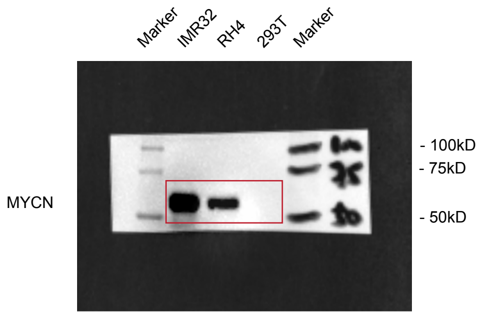

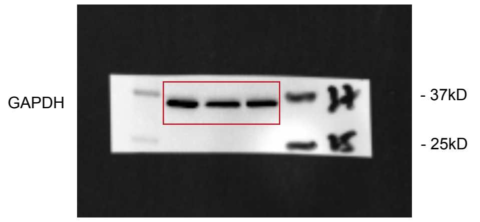

Raw immunoblots

Related to Fig. S2B

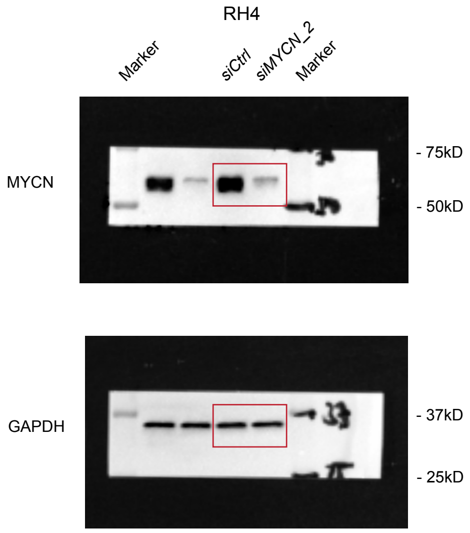

Raw immunoblots

Related to Fig. S4A

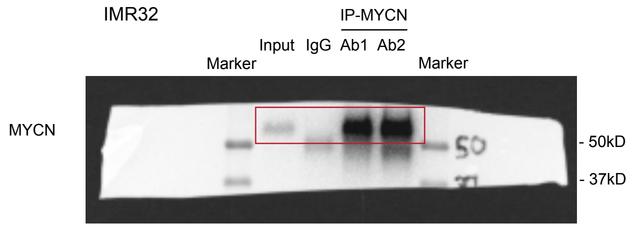

Raw immunoblots

Related to Fig. S4D

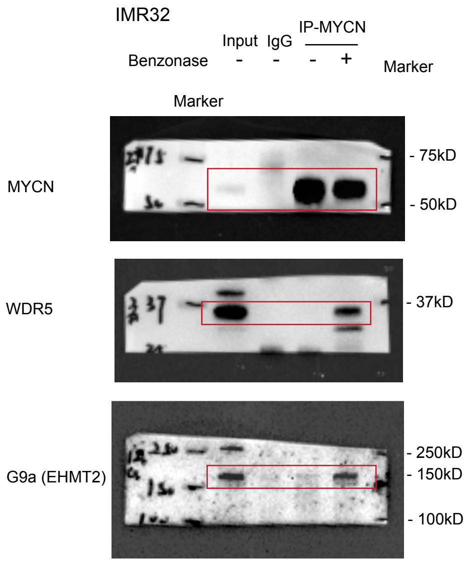

Raw gel

Related to Fig. S6E

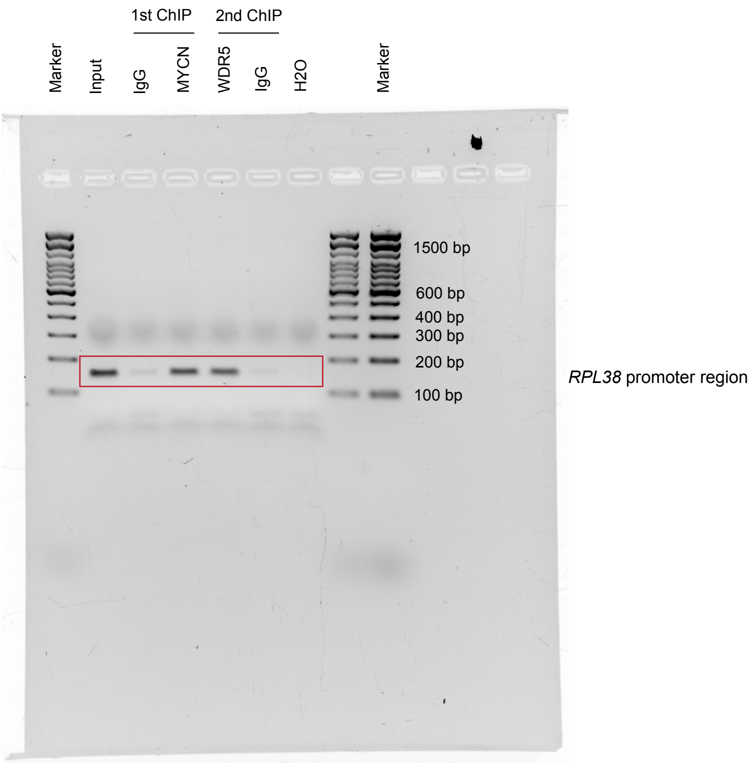

Raw immunoblots

Related to Fig. S7A

Related with Fig. S7A, the left panel

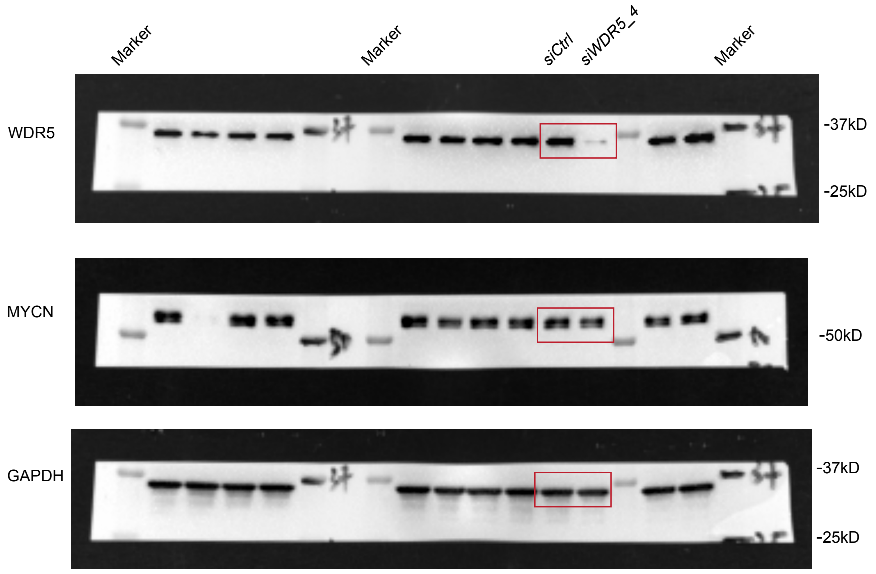

Related with Fig. S7A, the right panel

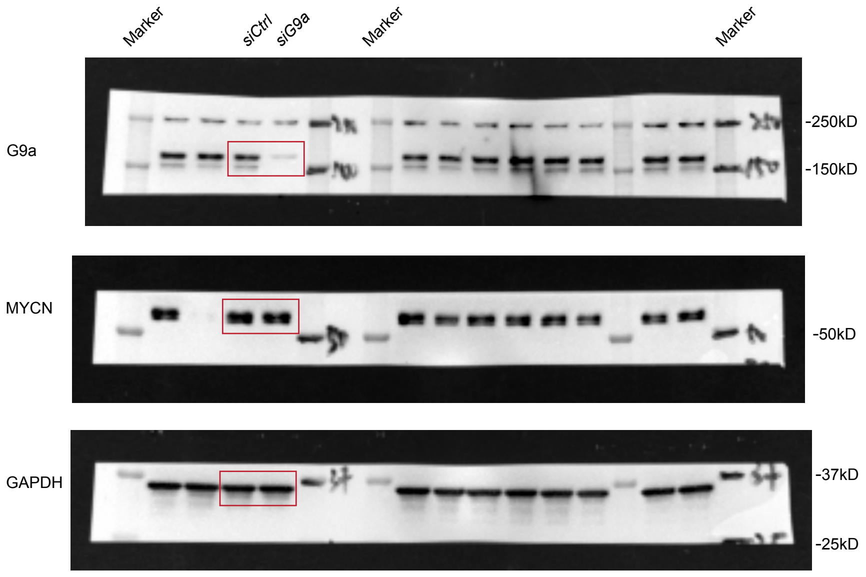

Supplement: S1 Raw Images — (PDF) [file pbio.3002240.s021.pdf]
